# Developing Polymer Semi-Solid-State Gel Electrolyte with High-Performance Aqueous Zn-Mn Battery-Type Hybrid Capacitor Device for MnO_2_–MWCNT Cathode

**DOI:** 10.3390/polym18182184

**Published:** 2026-09-08

**Authors:** Vediyappan Thirumal, Perumal Rajivgandhi, Jinho Kim

**Affiliations:** 1 School of Mechanical Engineering, Yeungnam University, Gyeongsan 38541, Republic of Korea; 2 Department of Chemistry, Nehru Memorial College, Bharathidasan University, Puthanampatti, Trichy 621 007, Tamil Nadu, India

**Keywords:** hybrid capacitor, zinc–manganese, hybrid battery, solid-state electrolyte

## Abstract

In recent years, energy storage devices have had a lower energy density for supercapacitor devices. Fortunately, certain drawbacks limit the liquid-based battery-type aqueous zinc-ion hybrid capacitor electrodes. For this reason, zinc–manganese (Zn-Mn)-based zinc-ion hybrid supercapacitors (ZIHSCs) have been designed using a manganese-dioxide-functionalized carbon nanotube (MnO_2_–f-MWCNT) battery-type cathode in a semi-solid gel–free-standing film electrolyte. Herein, as-prepared MnO_2_–MWCNTs are synthesized and assembled for nanostructured cathode composite materials by a facile hydrothermal technique. In this work, MnO_2_ nanorods with f-MWCNTs are applied to the electrode, resulting in a semi-solid-state gel film electrolyte realized by assembling the Zn-Mn hybrid capacitor. The materials’ physical–chemical conformation and their unique characteristics, crystalline structures, and different morphologies are studied through XRD, FE-SEM, FE-TEM, and XPS analysis. In this work, the design of major-source MnO_2_-based materials for positive and battery-type zinc metal anode approaches, along with the electrochemical properties of MnO_2_–MWCNT//Zn hybrid charge storage mechanisms, are evaluated. The coin-cell-type ZIHSC investigation of cyclic voltammetric (CV) curves and lower constant current charge/discharge (GCD) and electrochemical impedance (EIS) methods is also carried out. In addition, the maximum specific capacitance values, 339.98 mAh/g and 203.52 mAh/g, were observed for MnO_2_–MWCNT//Zn and MnO_2_//Zn at 0.2 mA/g, respectively. Finally, the higher cycling stability of MnO_2_−f-MWCNT of a 94.15% capacity retention after 15,000 cycles was evaluated and compared to MnO_2_//Zn of 73.05% retention in ZIHSC device applications. The assessment of electrochemical MnO_2_ cathode-based Zn-Mn ZIHSC performance is applicable for future aqueous electrical energy storage devices.

## 1. Introduction

In recent years, the rapid growth of the global population and the increasing use of electronic devices have created a strong demand for efficient electrochemical alternative energy storage (EES) systems through higher energy densities and long-term stability [1,2]. The widely available technologies, metal-ion batteries (such as lithium-ion batteries) and commercial supercapacitors, make for promising EES systems [3,4]. These electrochemical supercapacitors are generally classified into two main categories: (i) electric double-layer capacitors (EDLCs) and (ii) pseudocapacitors [5]. In addition, EDLCs store energy through a non-faradaic charge accumulation process at the electrode–electrolyte interface, forming electric double layers (E_DL_) such as carbon-based nanostructured active materials like conductive activated carbon (AC), reduced graphene oxide (RGO), and carbon nanotubes (CNTs); these carbonaceous-type materials undergo a good reversible adsorption and desorption mechanism in EDLCs [6,7]. In contrast, pseudocapacitors store charge through fast and reversible limited faradaic redox reactions occurring at or near the electrode surface. Examples of pseudocapacitive materials are SnO_2_, RuO_2_, NiO, Co_3_O_4_, MnO_2_, and conducting polymers [8,9,10]. In addition, Alexey Tsyganov et al. clearly demonstrated that the MXene family, particularly WCT and binder-free Ti_3_C_2_T_x_ MXene films, exhibits high-performance energy storage behavior and significantly enhances the intercalation pseudocapacitance of electrochemical energy storage devices [11]. These conventional electrochemical supercapacitors typically have a relatively low energy density, whereas batteries offer a higher energy density but suffer from a lower power capability and limited cycle lifetime, and lower potential for sluggish operation [12].

Among the various EES devices, electrochemical supercapacitors and batteries have conventional technologies belonging to various types; the secondary battery, like some of the work reports in recent years, has focused on a higher concentration due to the higher stable capacity and energy density [13,14]. The symmetric hybrid supercapacitor, called supercapatteries, is represented by lithium-ion capacitors (LICs) and sodium-ion capacitors (SICs); these devices have some drawbacks due to the greater number of electrode Li/Na-ion pre-doping, called pre-lithiation/pre-sodiation, and also have complex fabrication processes in ambient conditions with a glove box used in air-sensitive organic electrolytes [15,16]. Among them, zinc-ion energy storage has emerged as a promising alternative candidate, yielding a relatively higher energy density, faster charge/discharge rates, greater safety, and a greater cycle life [17]. The key advantages of zinc are as follows: it is an abundant source and safer anode compared to Li, Na, and K metal batteries; it has a lower fabrication cost; and it can be used as a future alternative energy source for EV vehicles. The zinc-ion-based device comprises hybrid capacitors that are gradually receiving attention from battery-type electrodes, acting as a negative zinc metal foil (Zn^2+^) electrode and a supercapacitive-based electrode with carbon nanostructures [18].

The operating mechanism of zinc-ion hybrid capacitors (ZIHCs) indicates that zinc foil serves as the battery-type electrode, while the capacitive electrode is typically composed of carbon materials combined with metal-oxide composites [19,20]. Carbon nanostructures exploiting the synergistic effects between these two components are essential for enhancing the conductivity network electrode and improving the storage of the specific capacitance and energy density of ZIHCs [21,22,23]. In battery-type hybrid capacitors, the selection of cathode active materials is critical, such as transition-metal oxides or phosphates (e.g., MnO_2_, V_2_O_5_, MoS_2_, and FePO_4_) and MXenes [24,25,26,27]. Among them, zinc-ion storage in battery-type materials offers a high specific capacity and relatively high operating potential. Manganese-based materials, particularly manganese dioxide (MnO_2_), are therefore considered among the most promising battery-type cathodes [28,29]. Moreover, MnO_2_ exhibits a high specific capacity (>400 mAh/g), a favorable operating voltage (1.3–1.35 V vs. Zn/Zn^2+^), and additional advantages including natural abundance, low cost, facile synthesis, and environmental benignity [30]. Consequently, Mn-based materials are widely employed as positive electrodes in aqueous zinc-ion energy-storage systems. Subsequently, MnO_2_ exists in five main crystalline phases (α, β, γ, δ, and λ), each with distinct chemical and structural characteristics. One-dimensional tunnel-type α-MnO_2_ (~4.6 Å) and two-dimensional layered δ-MnO_2_ (~7 Å) structures are particularly favorable for the rapid insertion of hydrated cations, whereas β-MnO_2_ (1D tunnel) and spinel-type λ-MnO_2_ possess smaller accessible channels, leading to relatively lower surface pseudocapacitance [31,32]. Owing to their large interlayer spacing, layered MnO_2_ structures can accommodate hydrated cations such as K^+^, Na^+^, Zn^2+^, and Ca^2+^, thereby enabling efficient charge storage [33].

Another main concern is the use of organic and aqueous electrolytes as a critical component in zinc-ion capacitors, as they govern Zn^2+^ transport between electrodes during charge–discharge processes and operate in a different voltage window with effective electrode wetting for next-generation energy storage applications [34,35]. Aqueous liquid electrolytes, typically composed of zinc salts dissolved in water, are widely used, with ZnSO_4_ being the most common choice due to its low cost, high ionic conductivity, and good electrochemical stability [36,37]. However, ZnSO_4_-based aqueous electrolytes suffer from drawbacks such as electrolyte leakage, rapid drying, and interfacial salt accumulation, which can accelerate capacitance fading and deteriorate the rate performance [38]. The incorporation of MnSO_4_ as a supporting dual-electrolyte additive can effectively suppress Mn dissolution and enhance the electrochemical stability of MnO_2_-based electrodes. In addition, the presence of Mn^2+^ ions promotes reversible redox reactions and improves ionic transport kinetics, resulting in an enhanced pseudocapacitive performance and cycling durability. To address these issues, recent studies have focused on semi-solid gel electrolytes based on monomer or polymer matrices that form three-dimensional gel networks. Typical polymer hosts include poly(vinyl alcohol) (PVA), polyacrylamide (PAM), gelatin, sodium alginate (SA), and poly(vinylidene fluoride-co-hexafluoropropylene) (PVDF-HFP) [39,40,41]. Among these systems, ZnSO_4_ combined with sodium-alginate-based cross-linked hydrogel electrolytes exhibits excellent mechanical strength, low-temperature tolerance, and stable ionic transport. Compared with conventional aqueous electrolytes, solid hydrogel electrolytes provide a balanced and effective strategy for achieving reversible zinc metal anodes [42,43,44].

In this work, we report a facile hydrothermal preparation of MnO_2_-decorated multiwalled carbon nanotubes (MWCNTs) for aqueous energy storage applications. Additional comparative materials include the first-step preparation of MnO_2_ nanostructures and surface-functionalized multiwalled carbon nanotubes, used as electrode materials for zinc-ion aqueous-gel-based semi-solid gel electrolyte. In addition, the main composite electrode includes Zn^2+^ versus MnO_2_–MWCNT assembled coin-cell-type hybrid battery devices. The as-prepared materials MnO_2_, f-MWCNT, and MnO_2_–MWCNT composites were analyzed by FE-SEM, FE-TEM, and XPS surface characterization to successfully confirm the materials. The second key part of the aqueous electrolyte is 2 M ZnSO_4_ + 0.5 M MnSO_4_ salt system combined with a polymer-based semi-solid gel-electrolyte using poly(vinyl alcohol) (PVA) and polyethylene glycol (PEG). The battery-type zinc-ion hybrid supercapacitors (ZIHSCs) demonstrated potential ranges of 0.0–1.6 V (vs. Zn^2+^) through CV, EIS, and battery-type GCD curve electrochemical analysis. Finally, the electrochemical performance of the semi-solid gel ZIHSCs device and the related highlights and potential applications can be evaluated as a real-world future electrochemical energy storage device.

## 2. Experimental Section

### 2.1. Materials and Methods

This work method implemented an in situ hydrothermal reactor using manganese (II) nitrate tetrahydrate (Mn(NO_3_)_2_·4H_2_O) with a purity of 98%, purchased from Alfa Aesar (Ward Hill, MA, USA), used as a precursor in synthesizing manganese oxide nanostructures. Battery Grade 99%, Pure Multi-Walled Carbon Nanotubes (MWCNTs), Powder type (Diameter ~20 nm, Length 5 µm) were received from “Carbon Nanomaterials Technology Co., Ltd.” (Pohang-si, Republic of Korea). Moreover, Ethyl Alcohol (C_2_H_5_OH) and Deionized (DI) Water (extra-pure laboratory grade from “REAGENTS DUKSAN (Jincheon-gun, Republic of Korea), High purity chemicals”) were used for the entire in situ hydrothermal synthesis process. The concentrated pure sulfuric acid (H_2_SO_4_) 99% and nitric acid (HNO_3_) 90% were acquired from SIGMA-ALDRICH (Darmstadt, Germany). All chemicals were of pure analytical laboratory grade and, without any further purification, were used as received.

### 2.2. Functionalization of Multi-Walled Carbon Nanotubes (MWCNTs)

Surface functionalization of multiwalled carbon nanotubes (MWCNTs) was carried out using a mixed acid treatment consisting of concentrated nitric acid (HNO_3_) and sulfuric acid (H_2_SO_4_) measured in a 1:3 volume ratio. Briefly, 0.5 g of pristine MWCNTs was dispersed in the acid mixture and refluxed under magnetic stirring for 1 h, followed by intermittent ultrasonication in a water bath below 25 °C for 30 min to ensure uniform dispersion and effective functionalization. After the reaction, the resulting suspension was thoroughly purified by repeated centrifugation and washing with deionized water and ethanol until any acidic residues and amorphous carbon impurities were completely removed and a neutral pH (~7) was achieved. Then, the finally collected black powder sample, denoted as functionalized MWCNTs (f-MWCNTs), was collected by vacuum filtration and dried in an electric oven at 80 °C for 6 h. In addition, these chemically functionalized f-MWCNTs were utilized for MnO_2_–MWCNT composite preparation.

### 2.3. MnO_2_—Nanostructure Preparations

Manganese dioxide (MnO_2_) nanostructures with a rod-like morphology were synthesized via a controlled hydrothermal reduction process. In a typical procedure, 2 g of potassium permanganate (KMnO_4_) was first dispersed in 40 mL of deionized water under continuous stirring to form a homogeneous precursor solution. Subsequently, 0.4 g of sodium borohydride (NaBH_4_) was introduced gradually as a reducing agent. After complete dissolution and stabilization of the reaction mixture, 10 mL of 5 M hydrochloric acid (HCl) was slowly added to adjust the acidity and facilitate the formation of MnO_2_ through redox interaction. The resulting solution was then transferred into a 100 mL Teflon-lined stainless-steel autoclave, maintaining a total filling volume of ~70 mL. The sealed autoclave was heated at 160 °C for 8 h to allow the reaction to proceed in a closed hot-air oven chamber. Upon completion of the reaction, the system was naturally cooled to room temperature, and the obtained precipitate was collected by centrifugation and thoroughly washed multiple times with deionized water and absolute ethanol to remove residual ions and byproducts until a near-neutral pH (≈6–7) was achieved. The purified product was then vacuum-filtered and dried at 80 °C overnight to eliminate adsorbed moisture; the final product consisted of well-defined MnO_2_ nanorod structures. The as-prepared material was subsequently used for structural and morphological confirmation, as well as for the preparation of a composite material with f-MWCNTs.

### 2.4. Synthesis of MnO_2_–MWCNT Composites

**Scheme 1:**
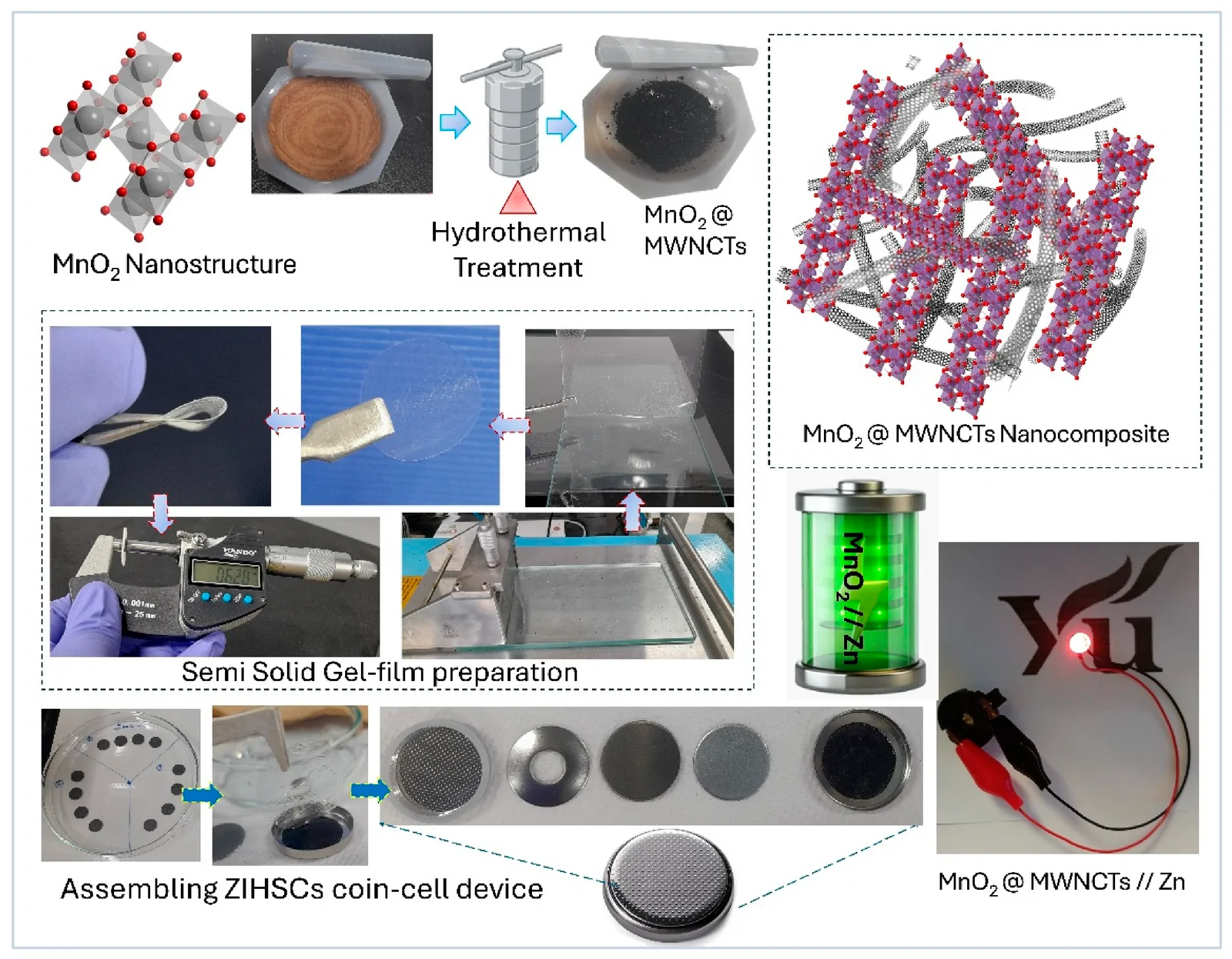
Schematic illustration of the experimental procedure for synthesizing MnO_2_ nanostructures and their composite formation with f-MWCNTs, along with representative sample photographs. Preparation steps for the semi-solid gel electrolyte films with assembly of the Zn-ion hybrid supercapacitor (ZIHSC) coin-cell device with LED illumination.

In typical synthesis of using a hydrothermal method for MnO_2_–MWCNT nanorod-like nanostructures, first, 1 g of as-prepared pure MnO_2_ powder sample and 0.25 mg of functionalized multi-walled carbon nanotubes (f-MWCNTs) were dispersed in a mixed solvent containing 40 mL of absolute ethanol. Then, 0.1 g of sodium borohydride (NaBH_4_) was added as the reducing agent and structure-directing agent, respectively. After that, 20 mL of DI water was added to the mixture. The MnO_2_ with f-MWCNT mixed precursor solution was stirred for 30 min. Then, this was followed by 15 min of water bath and sonication to obtain a uniformly distributed mixture solution. The final MnO_2_ with f-MWCNT solution was then transferred into a 100 mL Teflon beaker. The hydrothermal reaction was carried out at 140 °C for 12 h in a closed oven chamber. After the stainless-steel autoclave reaction setup cooled to room temperature, finally, the fine MnO_2_–MWCNTs composite powder product was collected and washed three times with DI water and ethanol to remove residual impurities. The purified products, namely, pure MnO_2_ and functionalized MWCNTs, were used for further material characterization and electrode slurry preparation. The entire experimental process and electrode preparation device are presented in a schematic and photographs shown in Scheme 1.

### 2.5. Material Characterization

As-synthesized materials MnO_2_, f-MWCNT, and MnO_2_–MWCNT composites were identified for the crystalline structure and crystallographic orientation planes using X-ray diffraction (XRD) analysis, as well as to confirm successful composite formation through characteristic diffraction peaks. The X-ray diffraction (XRD) for the model “PAN-Analytical” X’Pert PRO instrument operates (PANalytical B.V., Almelo, The Netherlands) at 40 kV with 30 mA through Cu-Kα radiation (λ = 1.5406 Å), recorded at (2θ = 5° to 80°). Additionally, field emission scanning electron microscopy (FE-SEM) was performed on a HITACHI (S-4800) system (Hitachi High-Technologies Corporation, Tokyo, Japan) operated at an acceleration voltage of 10 kV, which was carried out to observe surface morphology, particle distribution, and microstructural features of the materials (Surface analysis results are provided in Figures S1 and S2 in the Supporting Information (SI). Micro-Raman spectroscopy was used to investigate the vibrational characteristics and bonding configurations measurement using a model (Horiba, XploRA, HORIBA Scientific, Kyoto, Japan), with the following properties—laser: 532 nm, spectral resolution: 1.4 cm^−1^, and depth resolution: 2 μm (Raman spectra data presentation and discussions SI, Figure S3). In addition, field emission transmission electron microscopy (FE-TEM) studies were executed to study surface morphology and interfacial contact at the nano scale; field-emission transmission electron microscopy (FE-TEM), Thermo Fisher Scientific, Waltham, MA, USA (TALOS F200i) basic model, operated at 200 kV. The X-ray photoelectron spectroscopy (XPS) was employed to find the surface elemental composition and chemical states using X-ray photoelectron spectroscopy (XPS); Thermo Fisher Scientific, Waltham, MA, USA, K-Alpha (Al-Kα) source system was equipped with ion source energies ranging from ion gun, energy range 100 to 4000 eV.

### 2.6. Fabrication of Electrode and Electrochemical Characterization of ZIHSCs

The electrochemical performance of MnO_2_//Zn, f-MWCNTs//Zn, and MnO_2_-MWNCTs were tested on the electrochemical workstation SP-200, BIO-LOGIC-Science instruments (Seyssinet-Pariset, France), with a two-electrode system of coin cells, CR-2032 model (Essential Science, Daegu, Republic of Korea) for zinc ion hybrid supercapacitors, and ZIHSC device. Moreover, an active material homogeneously mixed slurry was prepared from pure MnO_2_ and MnO_2_–MWCNT active material. Subsequently, Ketjen black conductive carbon (C-300), and a polymeric PVDF binder was mixed in an 80:10:10 weight ratio using an NMP solvent with a few drops to form a uniform viscous paste with consistent mass loading. The resulting slurry was uniformly coated onto graphite foil substrates and subsequently dried in an oven at 80 °C overnight. The circular disc electrodes with a diameter of 15 mm were punched using a battery-type electrode die cutter to achieve coin-cell-type devices and were assembled using the MnO_2_–MWCNT composite as the cathode. The MnO_2_ and MnO_2_–MWCNT cathode active materials’ mass loading ranges of 1.8–2.4 mg ensured the specific electrochemical performance values. The formulation (Equations (S1)–(S3)) details have been provided in the Supplementary Information Section.

### 2.7. Preparation of Semi-Solid-State Aqueous Electrolyte

**Scheme 2:**
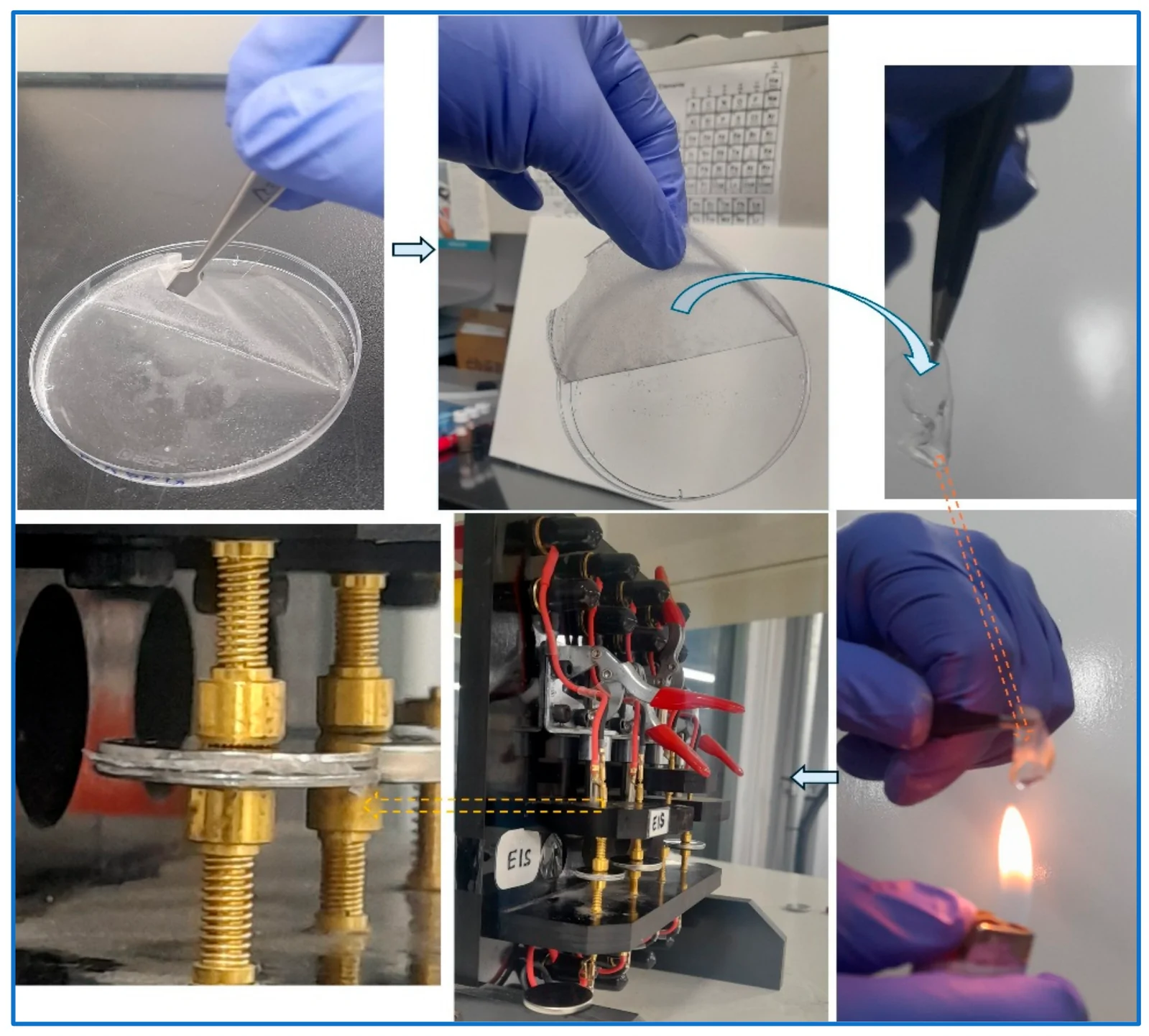
Photographic images for PVA–PEG gel film electrolyte with flame test, shown step by step, and gel-electrolyte film electrochemical tests.

The semi-solid-state aqueous gel electrolyte was prepared using a polymer-based approach to obtain a stable, gel-like electrolyte system. As a first step, prepare an aqueous liquid-based electrolyte containing 2 M ZnSO_4_ and 0.5 M MnSO_4,_ combined with a semi-solid gel electrolyte, which was prepared through a polymer-blending approach using poly(vinyl alcohol) (PVA) and polyethylene glycol (PEG). Initially, 8 g of PVA powder was gradually introduced into 50 mL of deionized water under continuous stirring and heated at 90 °C for 2 h to achieve complete dissolution and formation of a viscous, homogeneous solution. In a separate process, 0.2 g of PEG was dissolved in deionized water at 75 °C with constant stirring for 1 h to ensure uniform dispersion of the polymer chains. After confirming the full dissolution of both components, the PEG solution was slowly incorporated into the PVA matrix under mild heating conditions, followed by continuous stirring for approximately 20 min to promote uniform mixing and prevent phase separation, resulting in a stable gel-like electrolyte. The obtained semi-solid mixture was then cast into thin films using the doctor’s blade technique, employing a blade height of approximately 25 μm under semi-automatic coating conditions to ensure controlled thickness and uniform film formation. Then, homogeneous mixing and effective PVA–PEG of multivalent Zn^2+^ and Mn^2+^ ions within the polymer matrix were performed. In addition, the resulting homogeneous film of semi-solid gel electrolyte was poured into a glass plate using the doctor’s blade method and gradually dried at 50 °C overnight. After drying, a flexible semi-transparent gel film was easily peeled off and manually cut into circular discs with a diameter of 19 mm (CR-2032). Finally, a coin-cell assembly was used in zinc-ion hybrid capacitor devices, serving a dual function as both an electrolyte and separator. The prepared dual ions like a semi-solid gel electrolyte demonstrated a soft, jelly-like thin film texture and a flame test with a two-probe test model shown in Scheme 2. The thickness of the gel electrolyte film was measured to be ~0.632 mm using a digital screw gauge.

### 2.8. Assembling of Zinc-Ion Hybrid Supercapacitor (ZIHSC) Coin-Cell Device

**Figure 1:**
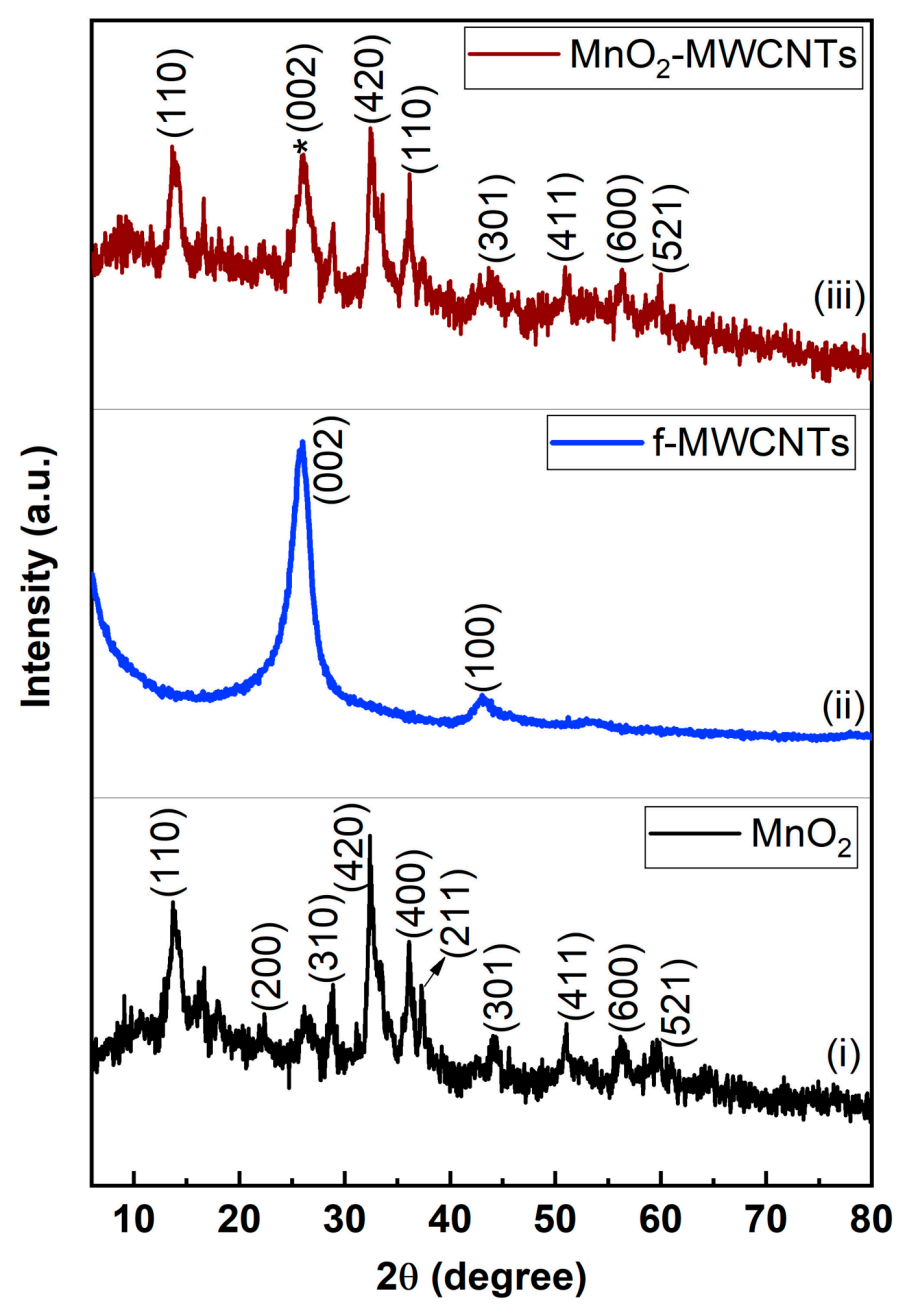
XRD pattern of (**i**) pure MnO_2_, (**ii**) f-MWCNT, and (**iii**) MnO_2_–MWCNT nanocomposite (inset: star symbol (*) is attributed to the carbon peak of the MWCNTs).

The coin-cell-type zinc-ion hybrid battery-type hybrid supercapacitor was assembled using high-purity Zn metal foil (99%) as the anode, as shown in Figure 1. A polymer-based semi-solid gel electrolyte consisting of 2 M ZnSO_4_ and 0.5 M MnSO_4_ was employed to enhance ionic conductivity and suppress zinc dendrite formation. Photographs of the PVA–PEG semi-gel electrolytes are presented in Scheme 2. Electrochemical tests were assessed using cyclic voltammetry, galvanostatic charge–discharge, and potentiostatic electrochemical impedance spectroscopy (EIS) in a range of 100 kHz–1.0 Hz and a potential amplitude of 10 mV. The CV and GCD profiles exhibited battery-type curves with redox behavior, confirming faradaic Zn^2+^ ion storage dominated by reversible insertion/extraction processes. All electrochemical measurements were conducted within a potential window of 0.0–1.6 V versus Zn^2+^/Zn for efficient energy storage characteristics. In addition, the surface morphologies of the Zn–Mn(SO_4_)–PVA–PEG semi-gel electrolyte film and the pristine PVA–PEG gel electrolyte were comparatively investigated using FE-SEM images.

## 3. Results and Discussion

### 3.1. XRD Analysis

The crystallinity and phase identification of the as-prepared MnO_2_ nanorod, functionalized MWCNT, and MnO_2_–MWCNT composites were examined (Figure 1) by XRD in the 2θ range of 10–80°. The diffraction pattern is 13.76° (110), 18.17° (200), 28.91° (310), 36.18° (400), 37.50° (211), 44.30° (301), 51.09° (411), 56.46° (600), and 59.80° (521), respectively. These diffracted values of pure MnO_2_ confirm the formation of a well-defined tetragonal α-MnO_2_ phase, indicating the high crystallinity of the nanorod structure. The observed diffraction peaks can be indexed to the characteristic crystal planes of α-MnO_2_, demonstrating a preferential orientation along specific lattice directions [45]. For pure f-MWCNTs, a strong and sharp diffraction peak appears at 25.92°, corresponding to the (002) plane, along with an additional peak at 43.02° indexed to the (100) plane. These peaks are associated with Sp^2^-hybridized carbon and confirm the well-crystalline hexagonal graphitic structure of the nanotubes. In the MnO_2_–MWCNT nanocomposite, the major diffraction peaks of MnO_2_ are retained at 13.61°, 32.46°, and 36.31°, indicating that the crystal structure of MnO_2_ is preserved after composite formation. The coexistence and slight merging of the MnO_2_ and CNT peaks suggest a strong interfacial interaction between the MnO_2_ nanorods and functionalized carbon nanotubes. In Figure 1, the XRD data confirm the successful hydrothermal growth of MnO_2_ nanorods anchored onto the CNT surface [46]. Such a well-crystalline and interconnected composite structure is highly favorable for Zn-ion transport and electrochemical stability. Overall, the XRD results indicate that the MnO_2_–MWCNT nanocomposite is a promising crystalline electrode material for Zn-ion energy storage devices.

### 3.2. FE-SEM Surface Analysis

**Figure 2:**
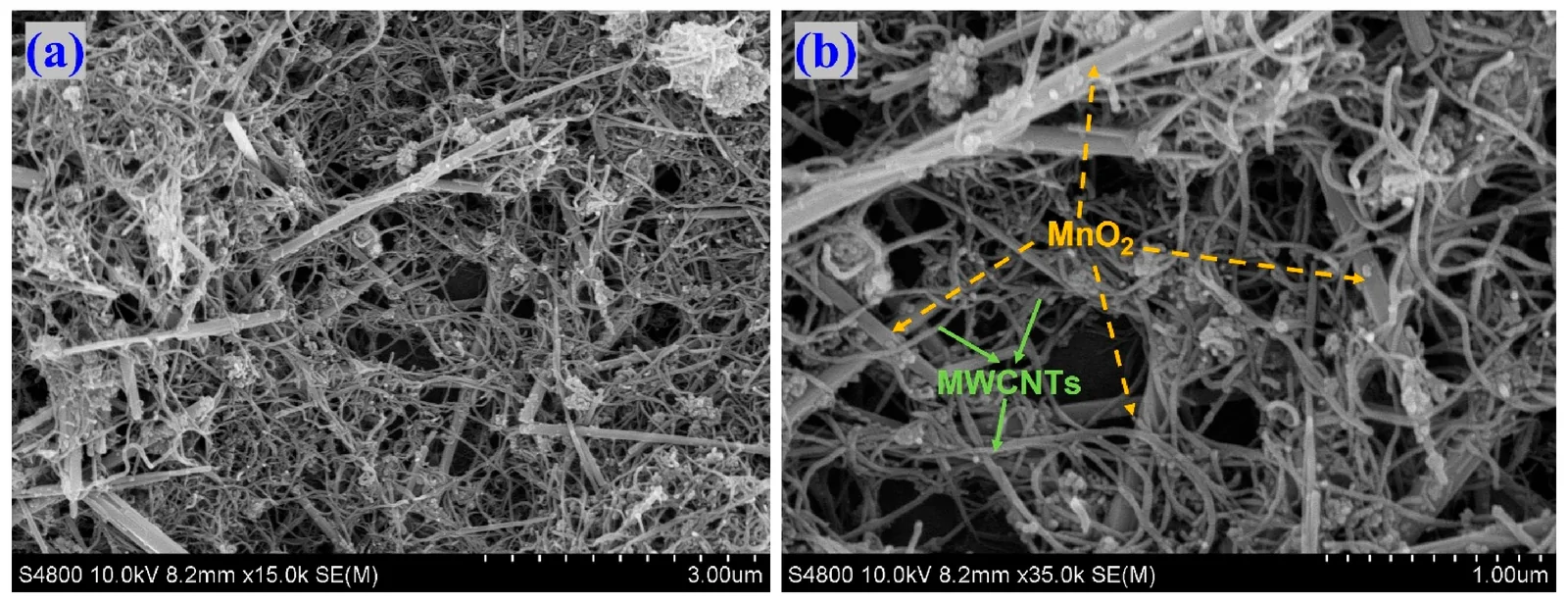
(**a**) FE-SEM surface morphological images of MnO_2_–MWCNT composite; (**b**) magnifications of the surface morphology, separately denoted in the inset, highlighting the MnO_2_–MWCNT composite, where MnO_2_ nanorods and MWCNT bundles are found.

The FE-SEM surface analysis of MnO_2_–MWCNT composites is presented in Figure 2a,b. Figure 2a reveals the microscale morphology, where well-dispersed MWCNTs are clearly observed together with MnO_2_ nanorods and small manganese oxide nanoparticles. These components form a dense and merged hybrid structure, indicating the strong interfacial contact between the nanotubes and nanorods. The composite exhibits a uniform distribution of MnO_2_ along the MWCNT framework. In Figure 2b, the SEM images further show ultra-long, curved, and circular CNTs with a beaded appearance. Moreover, MnO_2_ nanorods are seen to be uniformly anchored and coated on the surface of functionalized MWCNTs. Correspondingly, within composite materials, no impurity particles or irregular mesh-like structures are detected, confirming the purity of the composite. The highly interconnected CNT-MnO_2_ nanorod network provides one-dimensional pathways with efficient pathways for electron and ion transport, making it a promising electrode material for Zn-ion and zinc–manganese hybrid energy storage device applications [47].

The pristine samples of MnO_2_ nanorods and chemically functionalized multi-walled carbon nanotubes (f-MWCNTs) were analyzed. Figure S1a,b shows FE-SEM surface morphological images at different magnifications, confirming the formation of rod-like microstructures for the hydrothermally synthesized MnO_2_ nanorod structure. Additionally, Figure S2a,b presents the surface morphology of functionalized MWCNTs, revealing highly entangled, ultra-long nanotubes even after chemical functionalization, without noticeable defects or impurities. 

### 3.3. FE-TEM and Elemental Mapping Analysis

**Figure 3:**
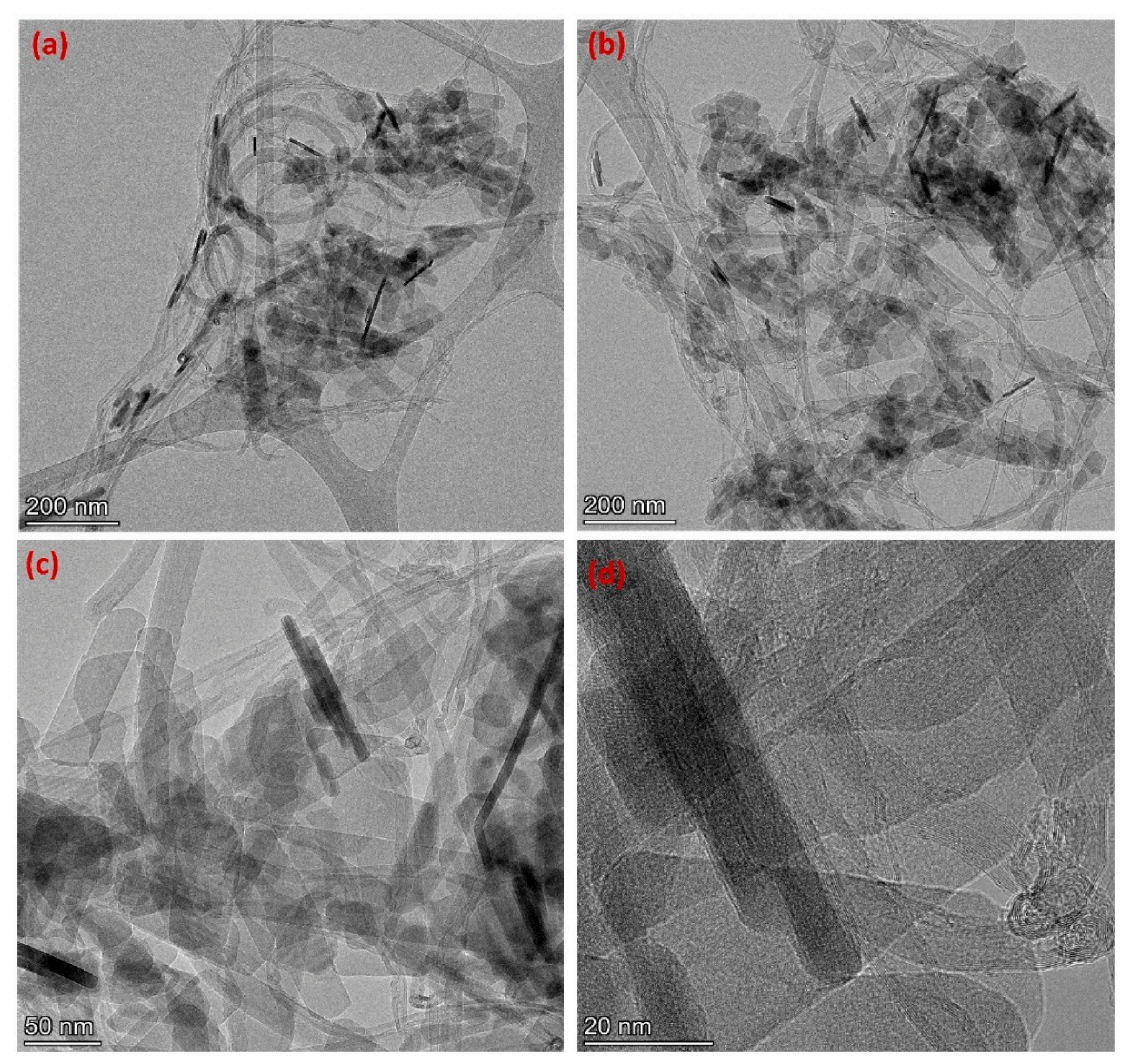
(**a**–**d**) FE-TEM surface morphological images of different magnifications of MnO_2_–MWCNT nanocomposites.

FE-TEM images of the MnO_2_–MWCNT nanocomposites (Figure 3a,b) recorded at a 200 nm scale reveal well-resolved MWCNT frameworks decorated with manganese oxide nanostructures. The TEM nano-graphs morphology clearly shows a circular and interlaced CNT network with a uniformly distributed MnO_2_ phase anchored on the functionalized MWCNT surface. The MnO_2_ appears predominantly as nanorod-like and flake-shaped structures, randomly yet homogeneously dispersed along the CNT walls, indicating an effective surface attachment. Higher-magnification TEM images provide deeper insight into the nanoscale morphology, confirming the intimate interfacial contact between MnO_2_ and the CNT matrix. As shown in Figure 3c, MnO_2_ nanorod-like flakes are strongly anchored to the f-MWCNT surface, forming interconnected and partially assembled rod networks. Figure 3d further illustrates the detailed morphology at higher resolution, where the CNT structure remains intact without observable surface damage after acid functionalization. The preserved CNT integrity and close contact with MnO_2_ suggest strong interfacial interactions [48]. The curved and closed surface edges of the MnO_2_–MWCNT composite confirm the successful hydrothermal growth and synergistic integration of MnO_2_ with the MWCNT conductive network.

### 3.4. TEM with EDS—Elemental Mapping Analysis

**Figure 4:**
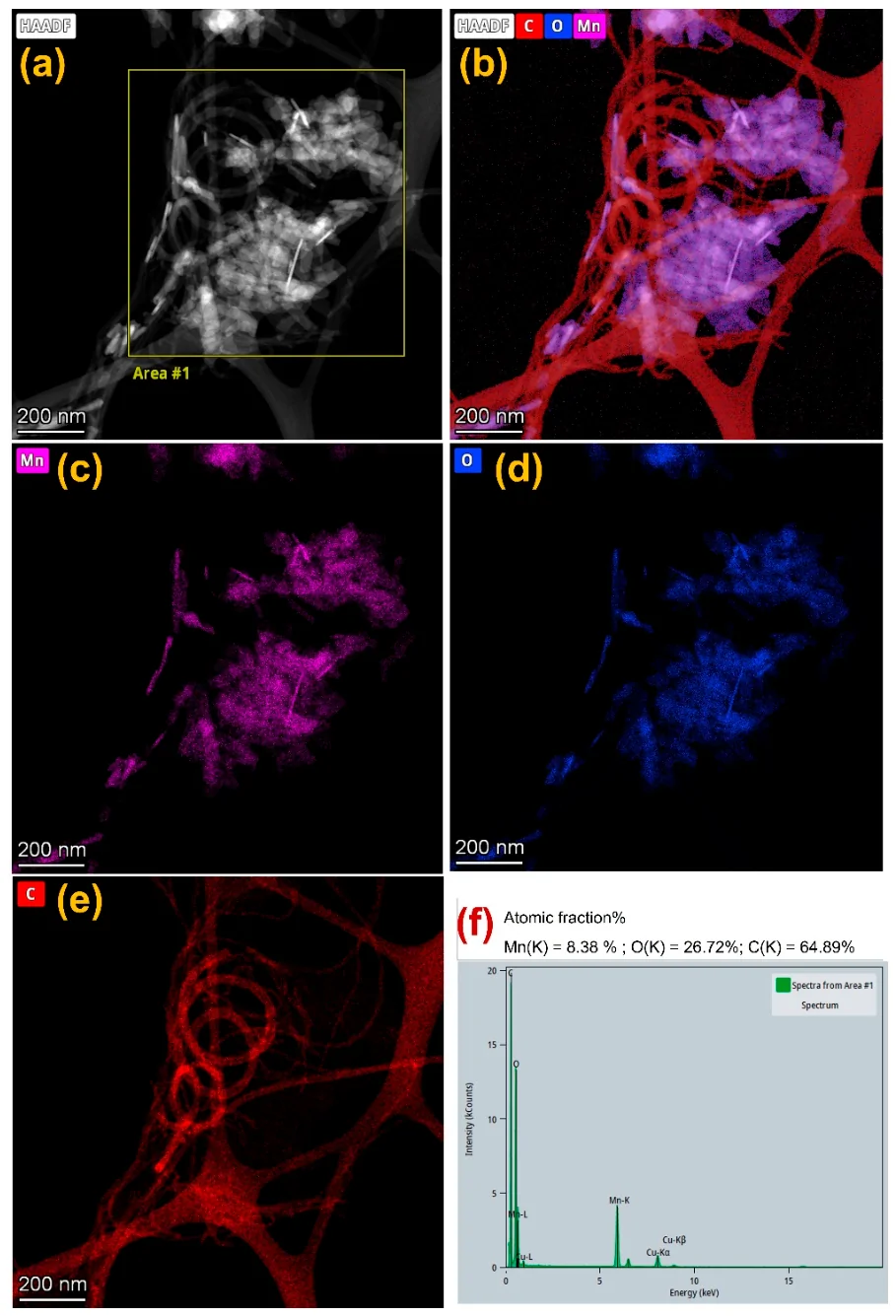
(**a**) FE-TEM surface images of MnO_2_–MWCNT composites: (**b**–**e**) overall mixed EDS color mappings for different elements; and isolated color matching for (**c**) Mn element, (**d**) O element (**e**), and C element, and (**f**) EDS spectrum graph with corresponding atomic% values.

Energy-dispersed spectroscopy (EDS) analysis was carried out using FE-TEM surface imaging to examine the elemental distribution in the MnO_2_–MWCNT nanocomposites. In Figure 4a, FE-TEM images show a well-defined composite surface morphology, accompanied by overall mixed EDS color mappings of the constituent elements. In addition, in Figure 4b–e, individual elemental maps clearly resolve the Mn, O, and C distributions, confirming a successful composite formation. The carbon element exhibits a circular red color mapping corresponding to the tubular framework of MWCNTs, consistent with the C1s EDS signal. The Mn and O elemental maps appear strongly overlapped, forming a rod-like dotted contrast that directly verifies the physical structure of MnO_2_ nanorods anchored on the carbon nanotube network. All elemental images precisely match the original TEM surface morphology, indicating uniform dispersion. The corresponding EDS spectrum in Figure 4f reveals approximate atomic percentages of Mn (K) 8.38 at.%, O (K) 26.72 at.%, and C (K) 64.89 at.%. Additionally, a thicker belt- or spider-like red dotted region is observed, which is attributed to the Cu–carbon mixed TEM grid.

### 3.5. XPS Analysis

**Figure 5:**
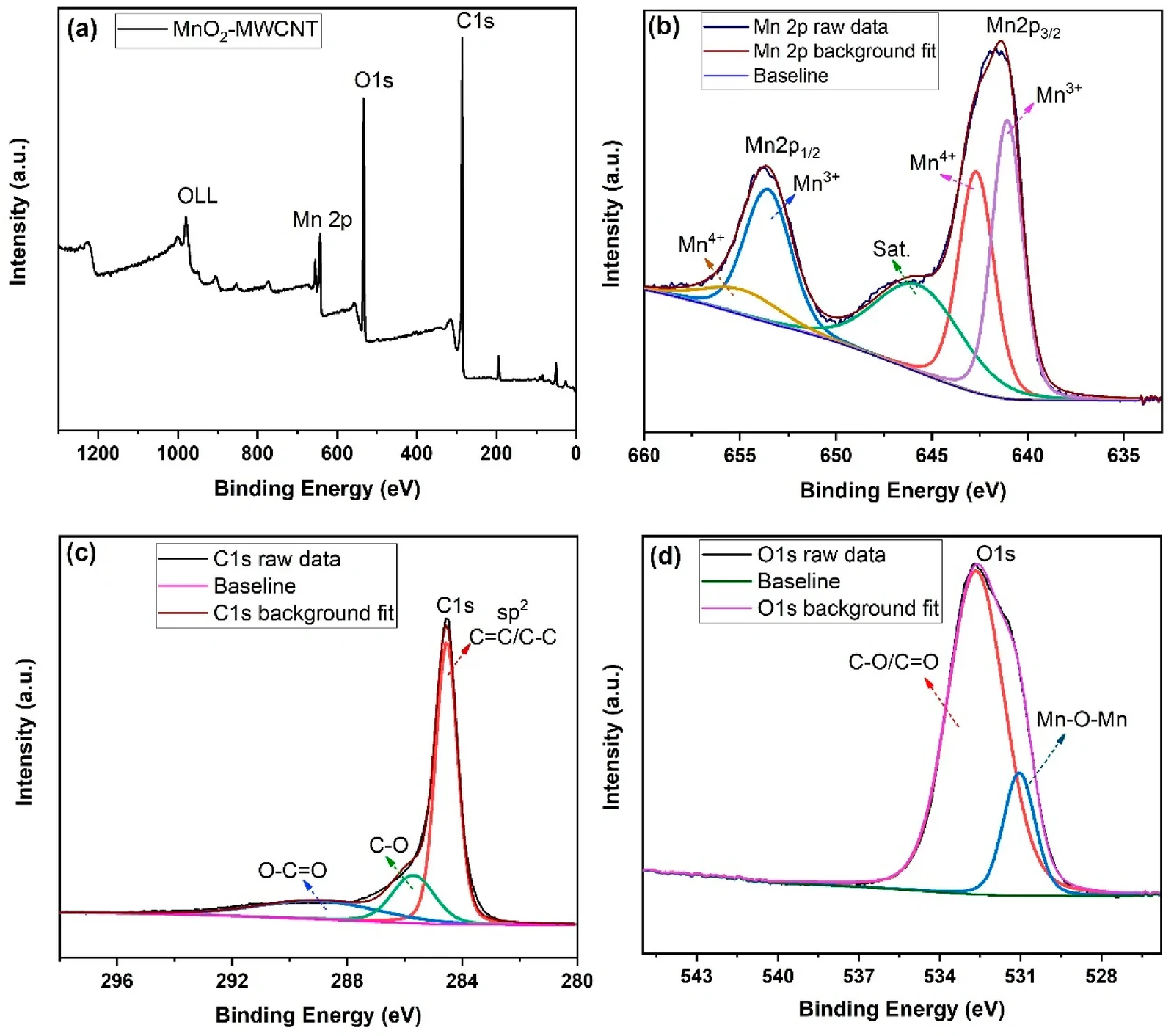
(**a**) XPS survey spectra of MnO_2_–MWCNT nanocomposites, (**b**) high-resolution deconvoluted spectrum of Mn 2p, and (**c**) C1s and (**d**) O1s fitted spectrum.

X-ray photoelectron spectroscopy (XPS) was employed to investigate the elemental composition, as well as the chemical bonding states of the as-prepared MnO_2_–MWCNT composite. The overall survey spectrum revealed, in Figure 5a, the presence of Mn, C, and O characteristic peaks corresponding to Mn2p (~640–660 eV), C1s (~284–288 eV), and O1s (~529–532 eV), respectively. High-resolution Mn2p spectra of individual elements were further analyzed by deconvoluted peak fitting using CASA-XPS software (version 2.3.26). Figure 5b shows the high-resolution Mn2p band discloses various well-defined fitted peaks and states assigned at 641.07 eV (Mn 2p_3/2_ of Mn^3+^), 642.69 eV (Mn 2p_3/2_ of Mn^4+^), 645.70 eV (satellite features), 653.55 eV (Mn 2p_1/2_ of Mn^3+^), and 656.12 eV (Mn 2p_1/2_ of Mn^4+^). The coexistence of Mn^3+^ and Mn^4+^ oxidation states indicates mixed-valence manganese oxide, which is beneficial for redox-active energy storage behavior. An XPS fitting showing Mn^4+^ as the dominant valence state means that the peak area corresponding to the higher binding energy of Mn^4+^ (2p_3/2_) (642.69 eV) is significantly larger than that of Mn^4+^ (2p_1/2_) (656.12 eV), reflecting their respective remaining valence d-electron configurations of 3d^3^ and 3d^4^. This indicates a mixed-valence manganese oxide where the surface is highly oxidized [49].

The quantitative XPS fitting results are summarized in Table 1. The Mn 2p_3_/_2_ (Mn^3+^) component at 641.19 eV is the dominant species with an atomic percentage of 32.01%, while the Mn 2p_3/2_ (Mn^4+^) peak at 642.87 eV contributes 23.03%, indicating a higher Mn^3+^ content. Similarly, the Mn 2p_1/2_ region shows 17.25% Mn^3+^ and 5.96% Mn^4+^, giving an overall Mn^3+^/Mn^4+^ ratio of approximately 1.55, which confirms the coexistence of mixed manganese oxidation states. This mixed-valence characteristic is consistent with non-stoichiometric manganese oxide and is expected to provide enhanced redox-active sites and improved electrochemical performance.

**Figure 6:**
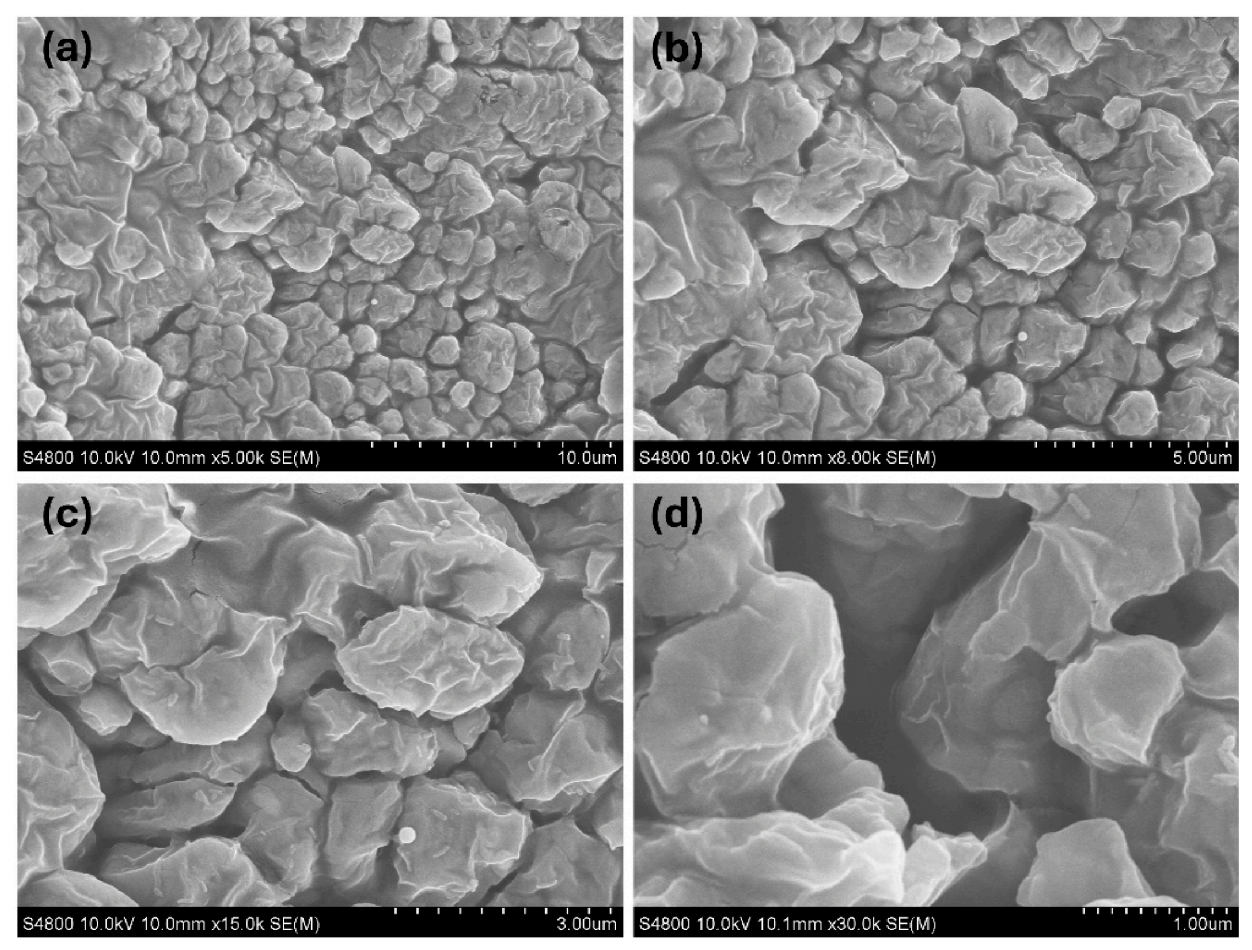
(**a**–**d**) FE-SEM surface morphological images of Zn-Mn(SO_4_)-PVA–PEG semi-gel electrolyte with different magnification.

Figure 5c shows that the high-resolution C1s spectrum exhibits three major deconvoluted peaks. The dominant peak at 284.54 eV matches C–C and C=C bonds, confirming the Sp^2^-hybridized carbon framework of the functionalized MWCNTs. Additional peaks at 285.69 and 288.98 eV are attributed to C–O and O–C=O bonds, respectively, arising from residual oxygen-containing functional groups such as hydroxyl and carboxyl moieties on the CNT surface [50]. Furthermore, in Figure 5d, the O1s-fitted XPS spectrum can be deconvoluted. The major peaks are the lower binding energy peak at 531.05 eV associated with Mn–O–Mn bonds in manganese oxide, while the higher binding energy peak and also O1s binding energy at 532.65 eV corresponds to C–O/C=O bonds originating from oxygen functional groups, as well as surface-adsorbed species due to air exposure and hydrothermal processing. Overall, the deconvoluted XPS spectra shown in Figure 6b–d provide clear evidence of strong interfacial interactions between MnO_2_ and f-MWCNTs, related to bonding energies within the composite structure. Such an intimate coupling is expected to facilitate the efficient charge transfer and structural stability, making the MnO_2_–MWCNT composite a capable electrode material for Zn–Mn hybrid device applications.

## 4. Semi-Solid-Gel Film: Surface Analysis and Electrochemical Properties

**Figure 7:**
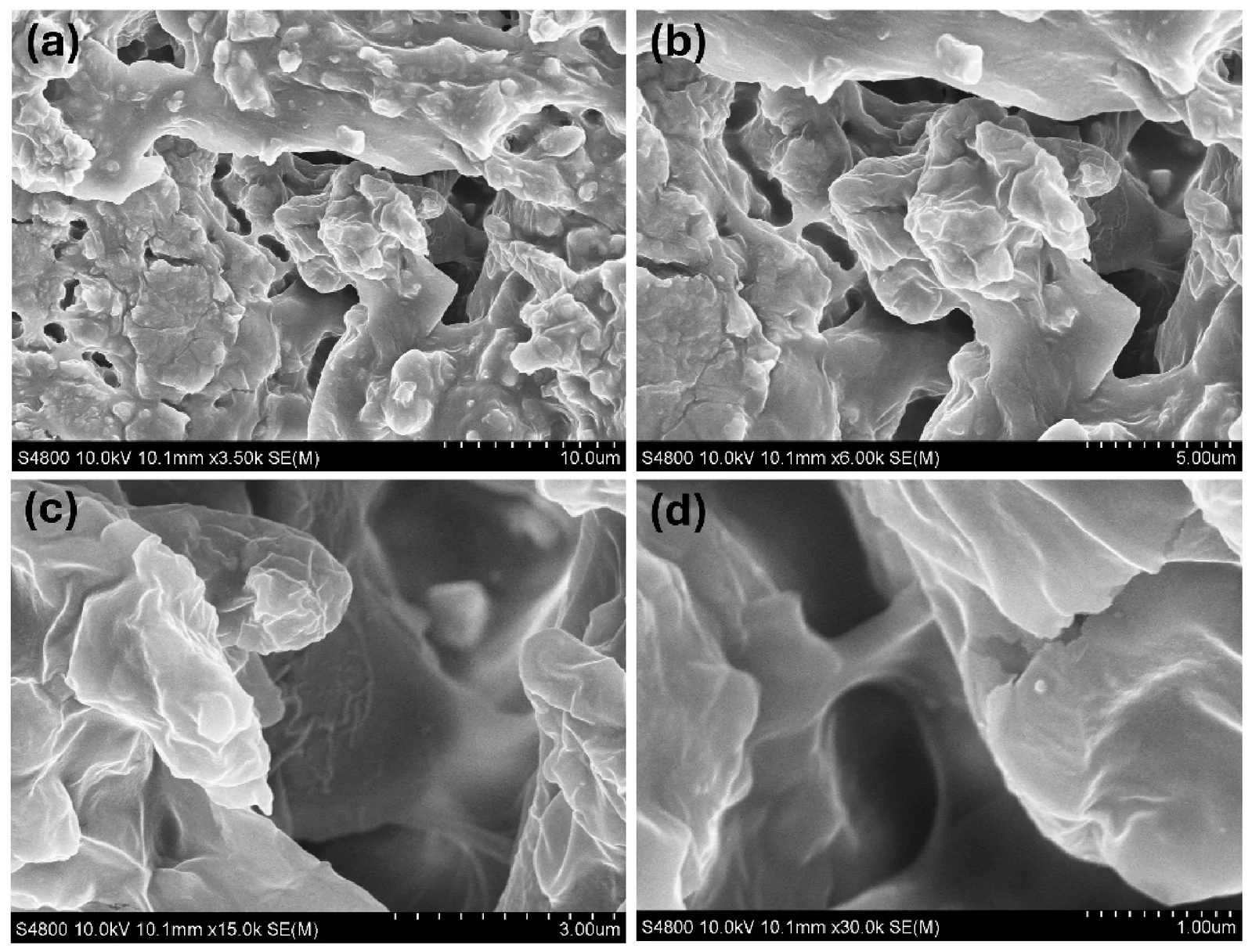
(**a**–**d**) FE-SEM morphological images of pure PVA–PEG semi-gel electrolyte with various enlargement surface structures.

The FE-SEM images of Zn–Mn(SO_4_)–PVA–PEG semi-gel electrolyte in Figure 6a–d show noticeable changes in surface morphology after salt incorporation. The lower-magnification images (Figure 6a,b) exhibit irregular and locally agglomerated surface structures with relatively uneven pores. At higher magnifications (Figure 6c,d), the surface displays more finely distributed microparticles and interconnected porous regions, indicating improved structural uniformity. These porous and interconnected surface features can provide favorable pathways for Zn^2+^ ion migration, supporting efficient ion transport and electrochemical performance in solid-state Zn-ion hybrid capacitor (ZIHC) devices. In comparison, Figure 7a–d shows the surface morphology of the pure PVA–PEG semi-gel electrolyte at different magnifications. At lower magnifications (Figure 7a,b; 10 and 5 μm), a relatively interconnected fibrous network with attached fine particles can be observed across the surface. The higher-magnification images (Figure 7c,d; 3 and 1 μm) clearly show the interconnected pores and void spaces formed by the PVA–PEG polymer network. The incorporation of the ionic salt promotes a more uniform arrangement of polymer chains and fine surface features, which can facilitate ionic transport within the semi-gel matrix.

**Figure 8:**
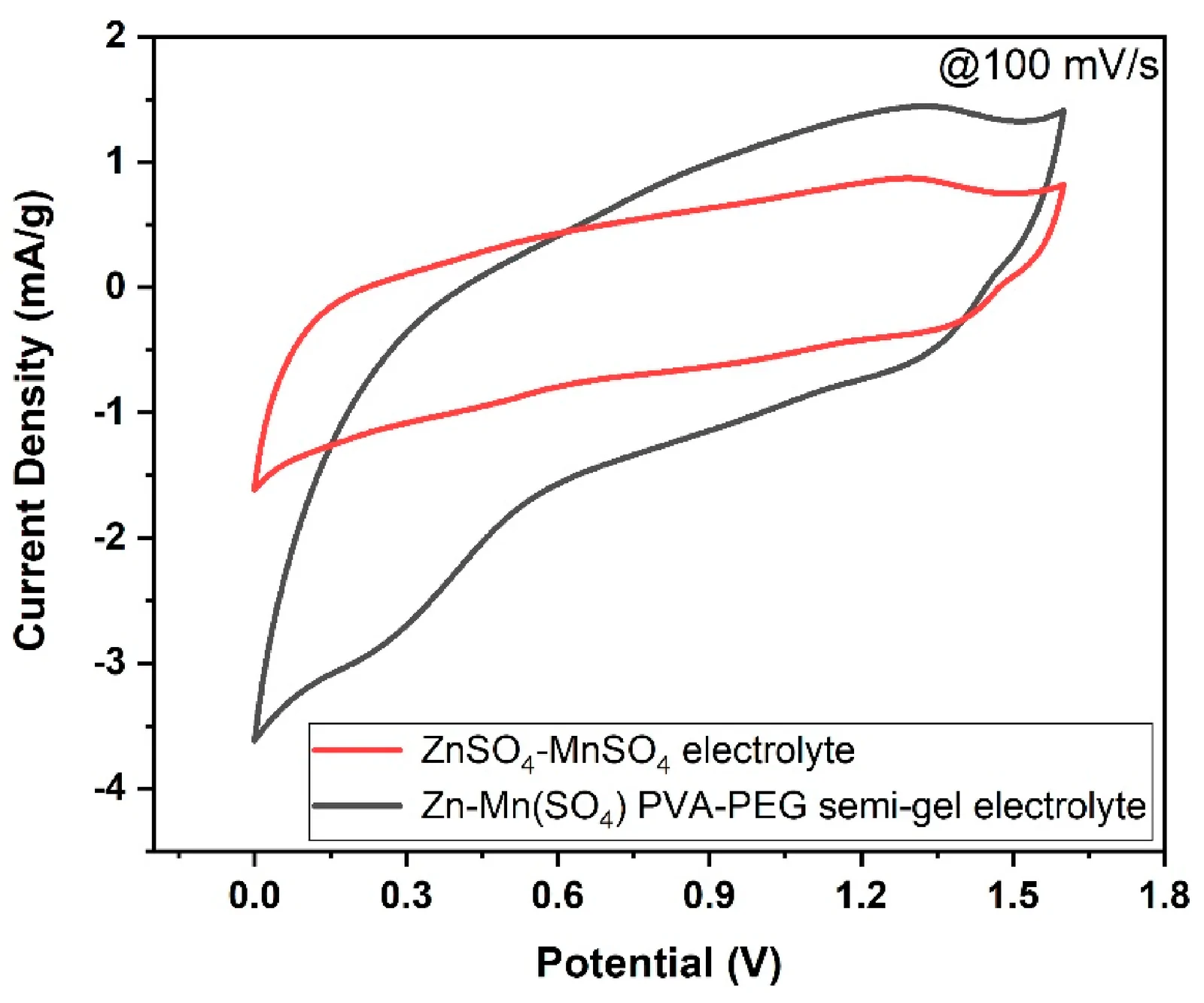
Electrochemical studies of cyclic voltammetry curves pure aqueous ZnSO_4_-Mn(SO_4_) electrolyte and compares Zn–Mn(SO_4_)–PVA–PEG semi-gel electrolyte film with MnO_2_–MWCNT electrodes.

**Figure 9:**
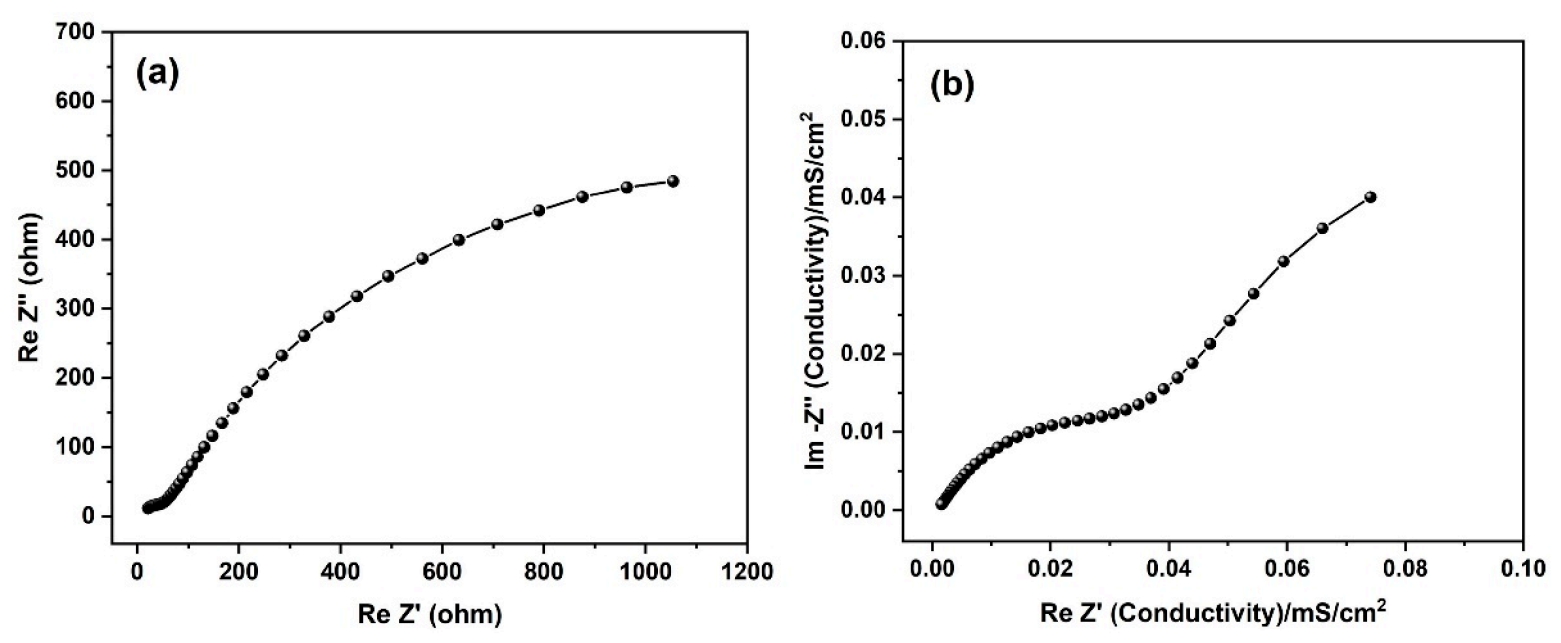
(**a**) EIS-Nyquist plot for pure aqueous ZnSO_4_-Mn(SO_4_) electrolyte; (**b**) ionic conductivity Cole–Cole diagram using EC-Lab software V11.60.

Figure 8 shows the cyclic voltammetry curves of MnO_2_–MWCNT electrodes using pure aqueous ZnSO_4_–MnSO_4_ electrolyte and Zn–MnSO_4_–PVA–PEG semi-solid gel electrolyte film. The comparison highlights the electrochemical behavior of the two electrolyte systems. The semi-solid gel electrolyte demonstrates a stable and suitable electrochemical performance. The EIS Nyquist plot in Figure 9a for the aqueous 2M ZnSO_4_–MnSO_4_ electrolyte shows a continuous impedance response with a low-frequency diffusion-controlled region. The relatively small high-frequency resistance indicates the ionic conduction of the aqueous electrolyte. The corresponding Cole–Cole plot in Figure 9b exhibits an average conductivity response over the measured frequency range. The real conductivity increases progressively from approximately 0.005 to 0.075 mS/cm^2^ as plotted, indicating effective ionic transport. From the plotted conductivity values, the average actual conductivity is ~0.04 mS/cm^2^. This relatively high ionic conductivity confirms the suitability of the ZnSO_4_–MnSO_4_ aqueous electrolyte for Zn^2+^ ion transport in ZIHC applications.

**Figure 10:**
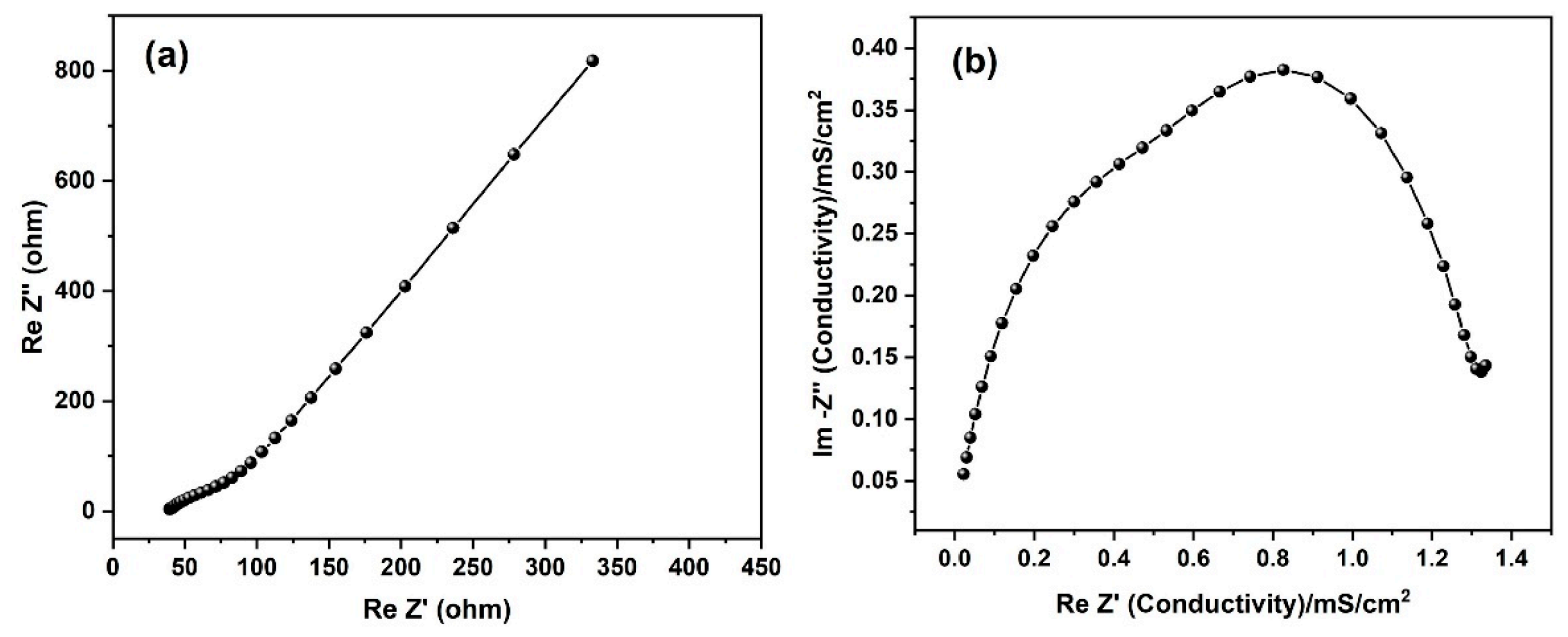
(**a**) (EIS) Nyquist plot and (**b**) ionic transport characteristics conductivity plot based on Cole–Cole analysis of pure aqueous ZnSO_4_-Mn(SO_4_) electrolyte.

The EIS response of the ZnSO_4_–MnSO_4_–PVA–PEG semi-gel electrolyte in Figure 10a shows an EIS Nyquist impedance spectrum, indicating effective ionic transport through the polymeric gel matrix. The corresponding Cole–Cole plot in Figure 10b exhibits a broad conductivity response, reaching a maximum of approximately 0.38 mS/cm^2^. Based on the plotted conductivity data, the average ionic conductivity is approximately 0.24 mS/cm^2^, indicating the good ion-transport capability of the semi-gel electrolyte. The enhanced conductivity is attributed to the hydrated Zn^2+^/Mn^2+^ ionic species and their efficient migration through the interconnected PVA–PEG polymer network. Thus, the ZnSO_4_–MnSO_4_–PVA–PEG semi-gel electrolyte provides a promising combination of ionic conductivity and solid-like structural integrity for ZIHC applications. For a comparison of Figure 9 and Figure 10, compared with the aqueous ZnSO_4_–MnSO_4_ electrolyte in Figure 10, which shows an average conductivity of approximately 0.04 mS/cm^2^, comparatively, semi-gel electrolyte exhibits a higher average value of ~0.24 mS/cm^2^. This enhanced ionic conductivity and self-supporting gel structure demonstrate that the PVA–PEG semi-gel electrolyte is a promising alternative to liquid electrolytes for future solid-state ZIHC devices.

## 5. Electrochemical ZIHSCs Performance

Zn–MnO_2_ battery-type electrodes were assembled into ZHC devices to evaluate the electrochemical behavior of the synthesized materials. A semi-solid, gel-like aqueous electrolyte was employed with an operating window of 0.0–1.6 V versus Zn^2+^/Zn. Although the aqueous gel electrolyte can extend the potential up to 1.8 V, the operation at a higher voltage caused the distortion and overlap of CV loops. Therefore, the aqueous-based ZHC full system was optimized and tested within a stable potential range from 0.0 to 1.6 V, to avoid the overpotential oxygen evolution reaction (OER) side reaction. Under this optimized window, the Zn–Mn hybrid supercapacitor exhibited well-defined and stable charge–discharge profiles. Reliable performance was maintained even at low current densities normalized to the active material mass loaded on the conductive carbon-foil substrate. 

### 5.1. Zinc-Ion Hybrid Supercapacitor MnO_2_//Zn

**Figure 11:**
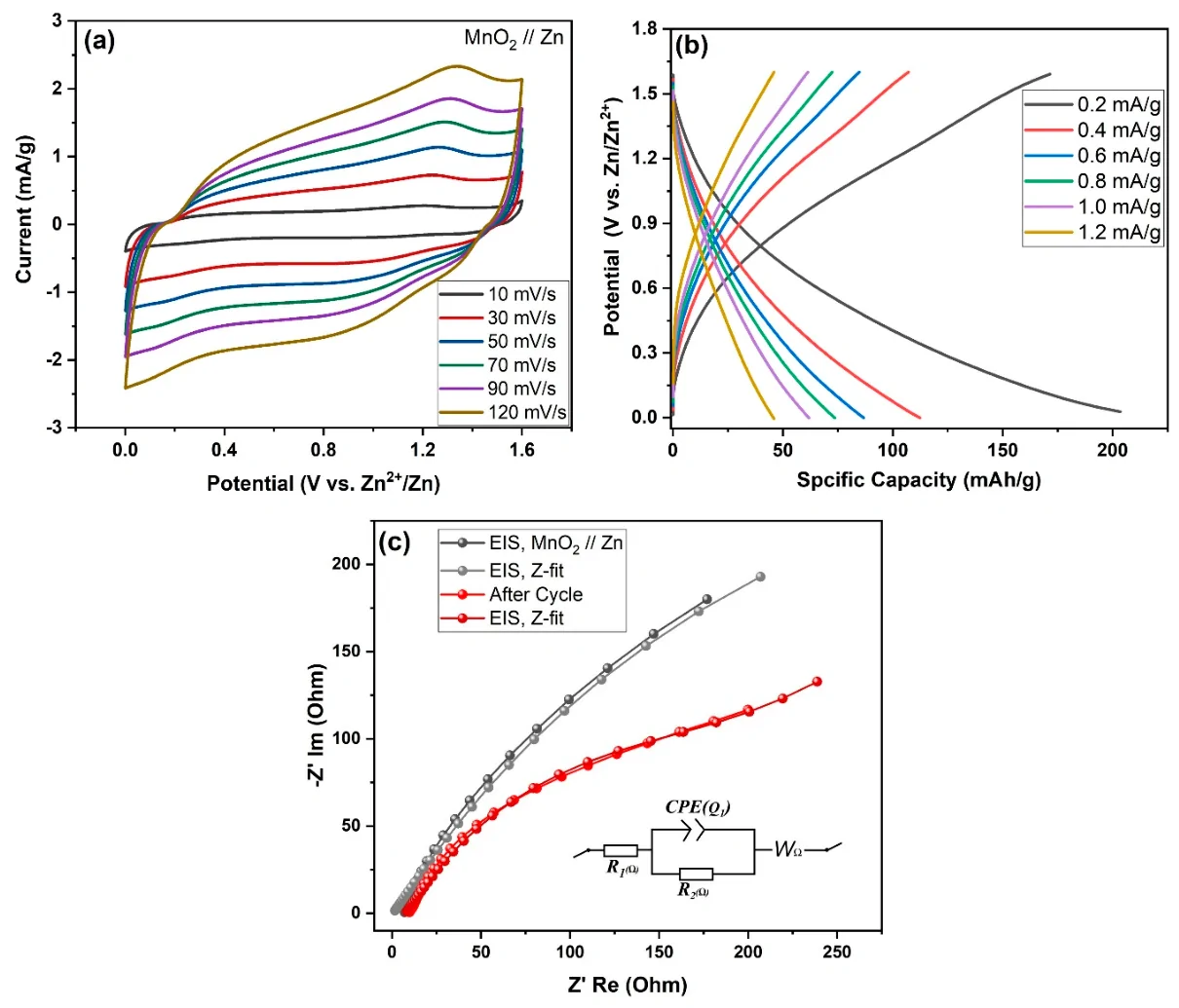
Electrochemical performance of the MnO_2_//Zn hybrid device showing (**a**) CV curves at different scan rates of 10–120 mV/s, (**b**) GCD battery-type profiles at various current densities 0.2–1.2 mA/g, and (**c**) impedance spectroscopy (EIS) Nyquist plot fresh and after cycling.

The electrochemical performance of the pure MnO_2_//Zn coin-cell device was assessed using CV, GCD, and EIS measurements, as presented in Figure 11a–c. The CV curves in Figure 11a, applied at scan rates of 10, 30, 50, 70, 90, and 120 mV/s, exhibit well-defined redox peaks with progressively enlarged enclosed areas as the scan rate increases, indicating enhanced electrochemical activity. This behavior confirms the effective Zn^2+^ ion storage within the MnO_2_ nanorod structure and reveals a mixed capacitive response arising from the interaction between nanostructured MnO_2_ and the Zn metal electrode. The aqueous electrolyte further facilitates the efficient ionic transport between the anode and cathode, contributing to improved electrochemical kinetics.

The pure MnO_2_//Zn Values of GCD curves shown in Figure 11b display battery-type profiles with a symmetric columbic efficiency of charge–discharge curves shapes at various constant current densities of 0.2, 0.4, 0.6, 0.8, 1.0, and 1.2 mA/g, corresponding to specific capacity values of 203.52, 112.28, 86.76, 73.59, 61.88, and 45.88 mAh/g, respectively. These results indicate pronounced battery curves with pseudocapacitive behavior even at low current densities, with combined good ionic transport and faradaic charge storage contributions in the MnO_2_ nanorod electrodes. The favorable performance of the MnO_2_ rod-like structures is attributed to the battery-type behavior of the Zn anode and the well-developed MnO_2_ nanostructure obtained via a facile hydrothermal synthesis route [51].

Figure 11c, EIS Nyquist plot of the fresh electrode, shows a low solution resistance (Rs) of about 4.67 Ω in the higher frequency part and negligible charge-transfer resistance, confirming good electrical conductivity. In the lower frequency section, a Warburg-type slope close to 45° is observed, which suggests low ion-diffusion resistance. After prolonged cycling, the EIS spectra reveal increased real-axis resistance and deviation from the ideal slope, indicating the structural deformation of MnO_2_ nanorods and possible SEI layer formation at the cathode interface, leading to partial surface degradation [52]. The EIS Nyquist parameter values are displayed in Supplementary Information (Tables S1 and S2). Overall, the Zn–Mn hybrid supercapacitor demonstrates stable ionic transport and reliable electrochemical performance when operated with a semi-solid gel polymer electrolyte membrane.

### 5.2. Zinc-Ion Hybrid Supercapacitor MnO_2_–MWCNT//Zn^2+^

**Figure 12:**
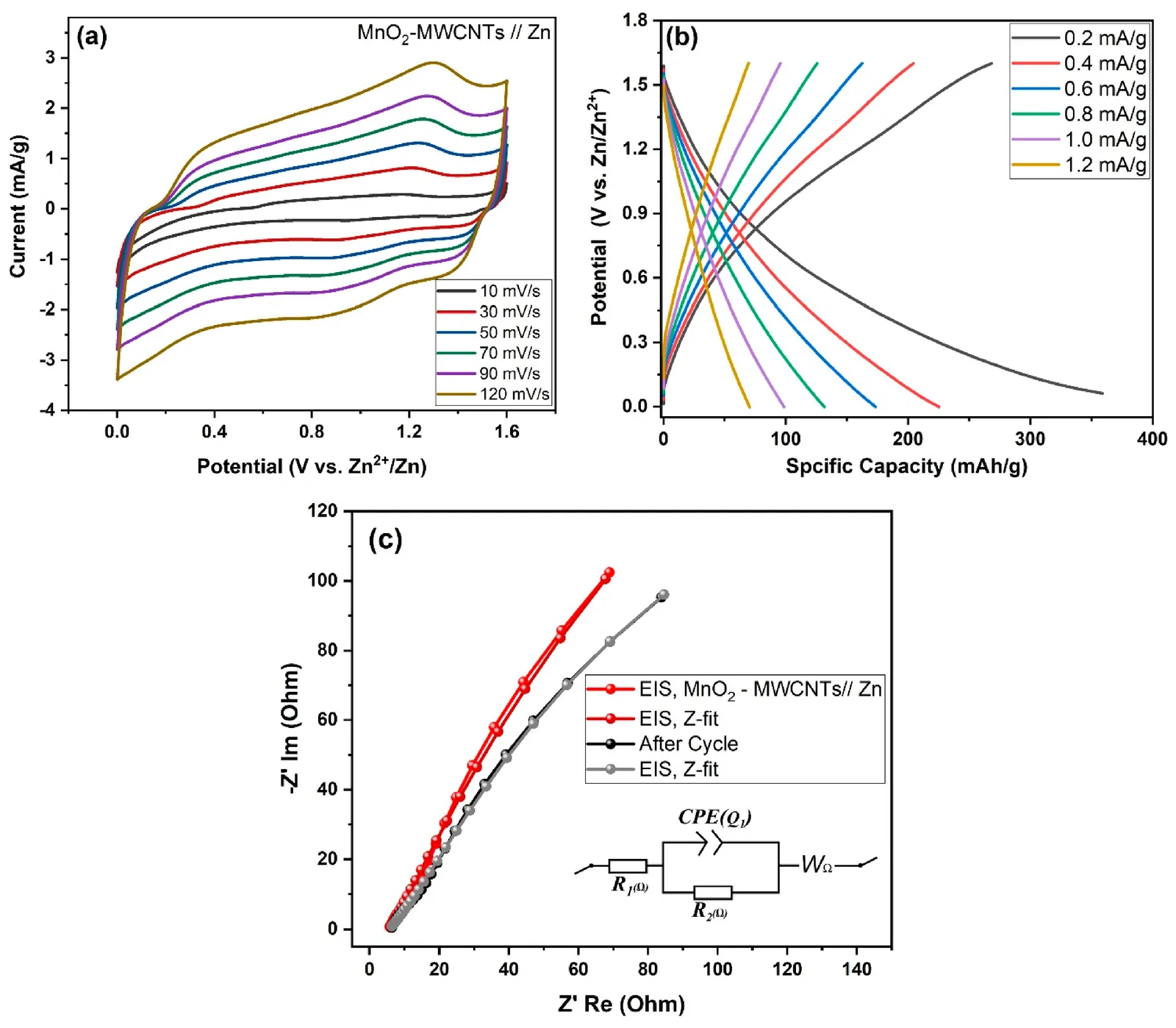
Electrochemical performance of MnO_2_//Zn and MnO_2_–MWCNT//Zn: (**a**) CV profiles at various applied scan rates (10–120 mV/s); (**b**) GCD curves at different current densities (0.1–1.2 mA/g); (**c**) Nyquist plot impedance comparison diagram before and after cycling (inset Randle’s circuit).

The MnO_2_–MWCNT electrode performance was evaluated electrochemically using a Zn-based coin cell configuration. Figure 12a presents the cyclic voltammetry (CV) curves of the MnO_2_–MWCNT electrode recorded at scan rates ranging from 10 to 120 mV/s. With an increasing scan rate from 10 to 120 mV/s, the CV curvatures show a gradual rise in current response while maintaining a similar partially redox shape, indicating enhanced electrochemical activity and a better rate capability. The enlarged enclosed area of the CV profiles suggests more complete Zn^2+^ insertion/extraction accompanied by reversible Mn^2+^/Mn^3+^ redox reactions, which is characteristic of battery-type behavior. The increase in the current density (mA/g) with the scan rate indicates a combined charge storage mechanism involving diffusion-controlled Zn^2+^ redox reactions and surface-controlled capacitive processes.

Figure 12b shows the galvanostatic charge–discharge (GCD) battery type curves to observe the capacity. The MnO_2_–MWCNT composite electrode delivers high specific capacities of 339.98, 224.92, 173.3, 131.47, 98.57, and 70.39 mAh/g at current densities of 0.2, 0.4, 0.6, 0.8, 1.0, and 1.2 mA/g, respectively. The high capacitance at low current density demonstrates the efficient utilization of active material and sufficient time for Zn^2+^ diffusion into the MnO_2_ structure. At low current densities, the electrode effectively accommodates multivalent Zn^2+^ and Mn^2+^ ion transport through a synergistic charge storage mechanism involving Zn metal stripping/plating at the anode and reversible redox reactions of MnO_2_ nanorods anchored on f-MWCNTs [53]. The combination of functionalized MWCNTs merits a role in enhancing the conductivity and ionic transport of electrochemical performance. The f-MWCNTs form a continuous conductive network within the MnO_2_ matrix, providing rapid electron transport pathways and reducing internal resistance. Additionally, surface functional groups on MWCNTs improve the interfacial contact between MnO_2_ nanorods and the electrolyte, facilitating faster ionic diffusion and more accessible redox-active sites. This interconnected conductive framework promotes efficient Zn^2+^ and Mn^2+^ multi-ionic transport through the aqueous gel-polymer electrolyte, leading to improved charge accumulation and storage kinetics [54]. Finally, comparative (Figure 11b and Figure 12b) specific capacity plots versus current density are presented in Figure S4.

The EIS Nyquist data analysis of the MnO_2_–MWCNT composite electrode is shown in Figure 12c. The EIS Nyquist plot was recorded in a frequency range from 100 kHz to 1 Hz to investigate the electrode–electrolyte interfacial properties. In the higher-frequency zone, the solution resistance (Rs) and charge transfer kinetics resistance (Rct) are measured to be ~5.4 Ω and 6.09 Ω, respectively, indicating lower resistance at the electrode/electrolyte interface and favorable charge transfer kinetics compared to the pure MnO_2_ electrode device. The EIS Nyquist plot parameter values are presented in the Supplementary File (Tables S3 and S4). The small semicircle diameter reflects efficient electron transport due to the conductive MWCNT network embedded within the MnO_2_ structure. In the lower-frequency region, the Warburg impedance displays a virtually vertical slope line, confirming good charge transfer behavior and rapid ion diffusion within the semi-solid-gel electrolyte solutions [55]. The presence of pseudocapacitive redox reactions along with electric double-layer capacitance (Cdl) further contributes to the overall charge storage.

**Figure 13:**
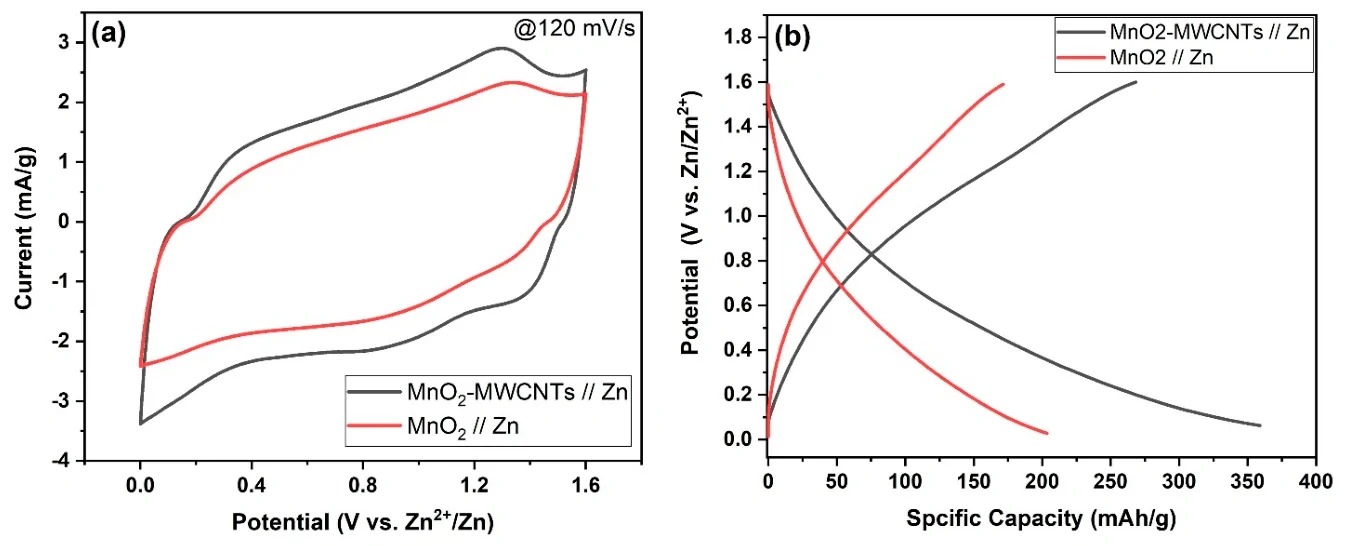
(**a**,**b**) Comparison graph of MnO_2_//Zn and MnO_2_–MWCNT//Zn composite devices.

The merged CV curves compared at a high scan rate of 120 mV s^−1^ reveal a significantly larger enclosed area for the MnO_2_–MWCNT device with pure MnO_2,_ shown in Figure 13a. Both devices operate within a similar electrochemical voltage window, indicating enhanced charge storage kinetics and a better rate capability; however, the MnO_2_–MWCNT electrode exhibits broader and more stable redox features, confirming stronger electrochemical activity. This improvement is attributed to the intimate attachment of MnO_2_ nanorods to the conductive MWCNT network, which promotes rapid ionic transport and efficient Zn^2+^ ionic diffusion [56].

Figure 13b shows comparative GCD curves recorded at a low current density of 0.2 mA/g, further highlighting the superior performance of the MnO_2_–MWCNT electrode. The battery discharge curve of the composite electrode is substantially longer than that of pure MnO_2_, 203.52 mAh/g at 0.2 mA/g, corresponding to a higher maximum specific capacity of 339.98 mAh/g at 0.2 mA/g for pristine MnO_2_–MWCNT composite electrodes in a ZIHSC device. The nearly symmetric charge–discharge profiles confirm improved reversibility and reduced polarization in the MnO_2_-CNTs composite system. The enhanced conductivity and electrolyte wettability introduced by functionalized MWCNTs facilitate the effective utilization of MnO_2_ active sites, as data evidence from XPS and TEM analyses shows, leading to improved electrochemical performance and stable hybrid charge storage behavior.

Overall, the higher electrochemical responses of the MnO_2_–MWCNT composite electrode device resulted in a higher specific capacitance and charge-transport kinetics in an aqueous semi-solid gel polymer PVA–PEG film electrolyte, which signifies the MnO_2_ nanorod CNT network active contributions from the surface redox reactions and also confirms the capacitance from the insertion/extraction of the assembling of coin-cell-type Zn versus MnO_2_ electrochemical device mechanisms beyond the generally reported zinc-ion capacitor. The manganese-based cathode materials, with enhanced multivalent ion Zn_2+_/Mn_2+_ charge storage, indicate Zn_2+_ pre-intercalated α-MnO_2_ (ZnxMnO_2_) nanorods in a blended aqueous electrolyte (2 M ZnSO_4_ and 0.5 M MnSO_4_ with PVA–PEG gel electrolyte) for stable Zn-Mn ionic storage [57]. These features make the MnO_2_–MWCNT composite electrode a promising candidate for high-performance Zn-ion hybrid supercapacitors and future energy storage applications.

**Figure 14:**
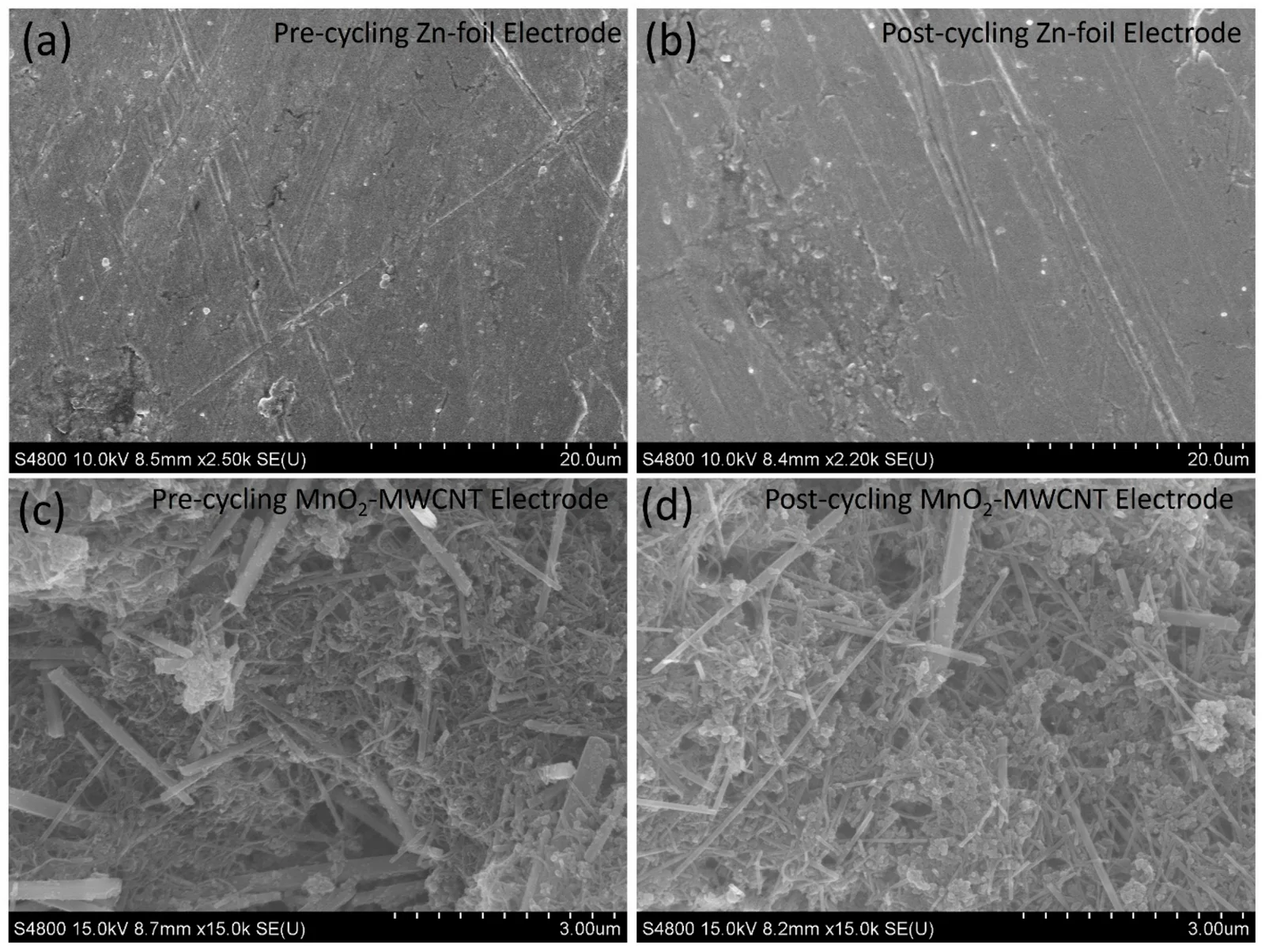
FE-SEM images of (**a**) Pre-cycling Zn-foil electrode, (**b**) post-cycling Zn-foil electrode, (**c**) Pre-cycling MnO_2_–MWCNT electrode, (**d**) post-cycling MnO_2_–MWCNT electrode of long-term cycling.

The pre-cycling and post-cycling FE-SEM surface morphological images of the Zn metal foil anode electrodes are presented in Figure 14a,b. Even after prolonged electrochemical cycling, the Zn anode maintained a relatively stable and smooth surface morphology with significantly reduced dendritic growth, which indicates the effective role of the gel polymer aqueous electrolyte system in suppressing zinc dendrite formation. In addition, Figure 14c,d shows cathode active material morphologies, and the fresh and post-cycling of the MnO_2_–MWCNT composite electrode. After long-term cycling, the MnO_2_ nanorod-like surface structure remains well-preserved with interconnected CNT bundle networks, demonstrating excellent structural integrity and electrode stability. These results confirm the robust electrochemical durability of both electrodes over 10,000 cycles. Therefore, the developed Mn–Zn hybrid system demonstrates strong potential as a promising candidate for advanced aqueous energy storage applications.

### 5.3. Cycling Stability Performance

**Figure 15:**
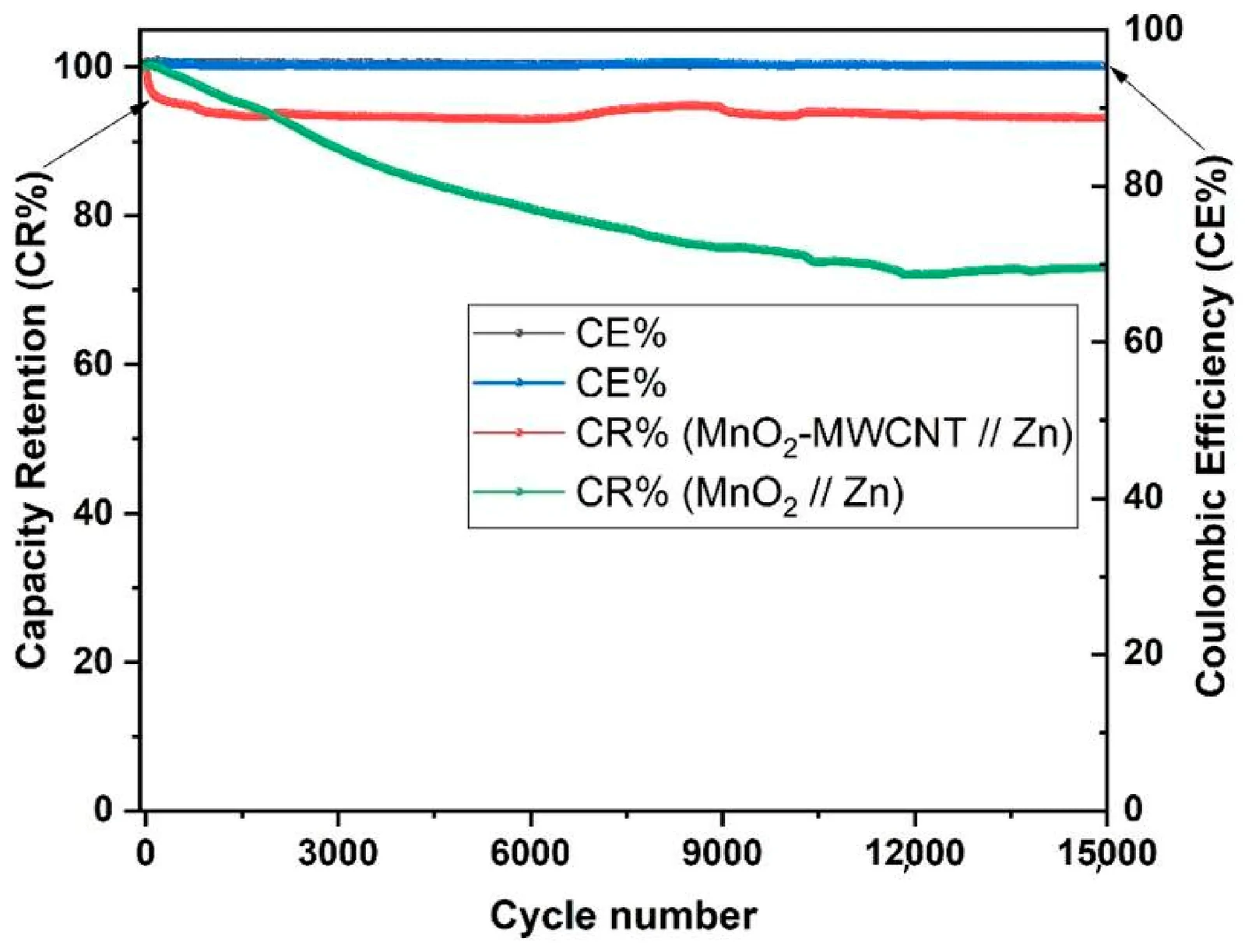
Cycling stability and coulombic efficiency of the MnO_2_–MWCNTs composite coin-cell device measured at 1.2 mA/g for 15,000 cycles.

The cycling stability of the coin-cell device was systematically evaluated to confirm the long-term durability of the electrode materials. The MnO_2_–MWCNT composite electrode exhibited excellent electrochemical stability when tested at a current density of 1.2 mA/g over 15,000 continuous charge–discharge cycles. The ZIHSCs hybrid coin cell device maintains highly stable charge–discharge profiles with an average coulombic efficiency of approximately 99.5%, indicating highly reversible electrochemical reactions. As shown by the red curve in Figure 15, the MnO_2_–MWCNT composite coin cell demonstrates superior cycling performance with a high energy efficiency of 94.15%. In contrast, a coin cell based on pure MnO_2_ electrodes shows a lower capacity retention of 73% after the same number of cycles. The significantly improved cycling for electrode active materials and the structural stability of the MnO_2_–MWCNTs composites are attributed to the well-integrated MnO_2_ nanorods and the conductive MWCNT network, which enhance the internal electron transport and structural integrity during repeated cycling. Finally, the before- and after-cycling FE-SEM surface images of the Zn anode and MnO_2_–MWCNT cathode after long-term cycling in high-performance zinc-ion energy storage systems are shown in Figure 15.

### 5.4. Specific Energy and Power Density Presentation

**Figure 16:**
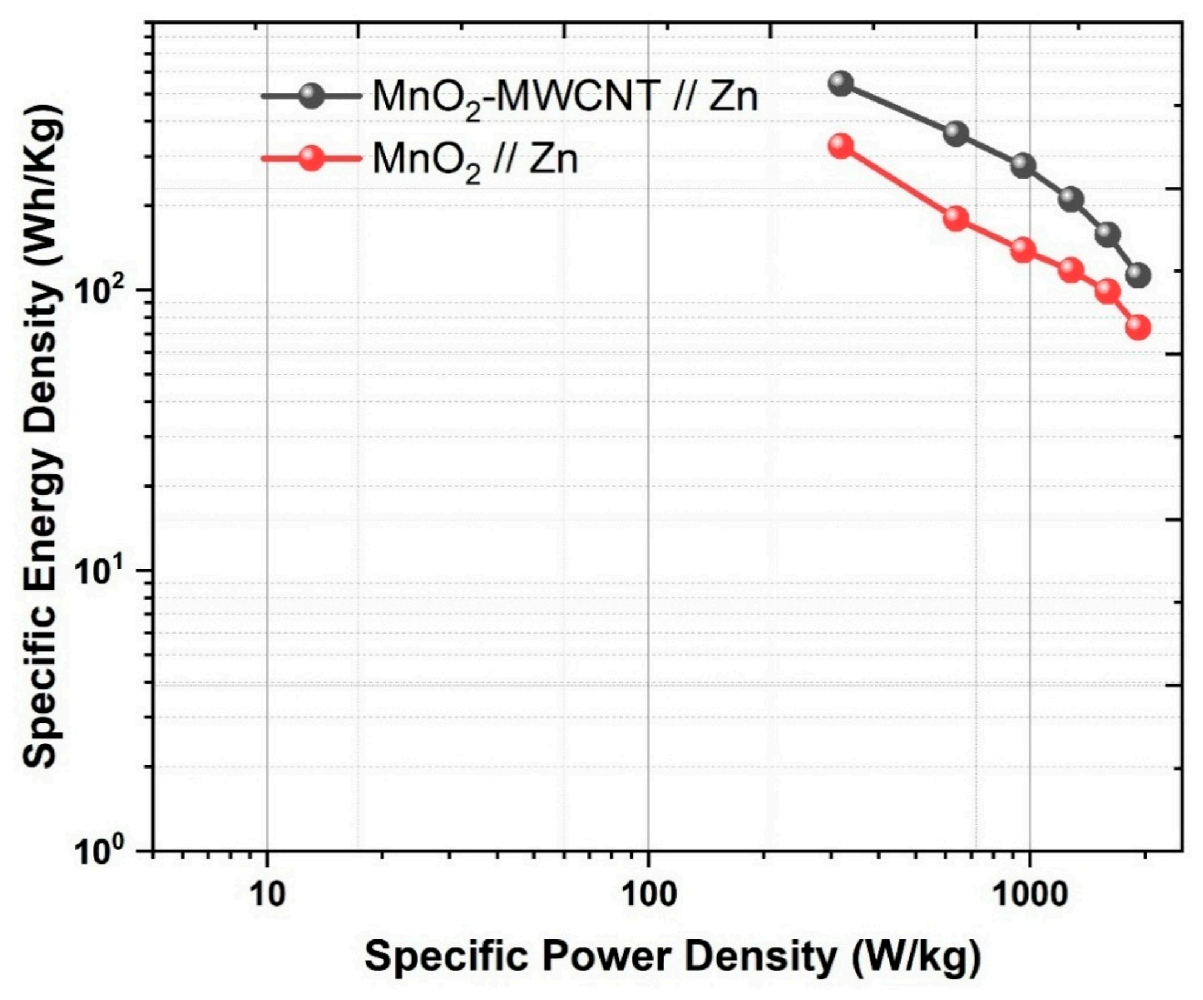
Ragone plots gravimetric plot data measured for ZIHSCs devices.

The energy and power density characteristics of the ZIHSC coin-cell device are shown in Figure 16. The pure MnO_2_-based ZIHC device delivers a high specific energy density of 99.01 Wh/kg at an applied current density of 1.0 mA/g, demonstrating an excellent energy storage capability. In addition, the comparatively better-response MnO_2_–MWCNT ZIHSC device achieves a maximum specific energy density of 157.71 Wh/kg in the Ragone plots. These energy and power density values represent the maximum performance obtained at a low current density, where a high specific capacitance is effectively utilized. In addition, the power density (PD) of 320 W/kg gradually increased with a maximum specific power density of 1920 W/kg, matching the applied lower current densities. Finally, a comparison with the recently reported Zn-ion hybrid capacitor devices are shown in Table S5 in the Supplementary Information (SI). The superior ED and PD performance is attributed to the battery-type MnO_2_ nanorod cathode combined with the conductive and wettable functionalized CNT network, which enhances the charge transport and electrolyte accessibility for developing future hybrid capacitor devices [58,59]. These results highlight the strong potential of the developed coin-cell device for future aqueous energy storage applications.

## 6. Conclusions

To conclude, the summary of this work has successfully developed a novel, high-performance Zn-MnO_2_ hybrid supercapacitor device with a semi-solid gel electrolyte. This work investigates a one-dimensional MnO_2_–MWCNT nanocomposite by a single-step hydrothermal approach. The structural analysis by XRD confirms that the synthesized MnO_2_ exhibits a well-defined tetragonal α-MnO_2_ phase. Further surface and morphological investigations using XPS and TEM verify the formation of MnO_2_ nanorod structures along with the presence of relevant functional groups and electronic states. FE-TEM combined with EDS elemental mapping demonstrates the uniform distribution of elements in both MnO_2_ and MnO_2_–MWCNT composite nanostructures, with atomic percentages of Mn (K) 8.38 at.%, O (K) 26.72 at.%, and C (K) 64.89 at.%.

The as-prepared electrode materials were successfully applied in a novel hybrid energy storage system employing a Zn–Mn electrochemical battery-type hybrid capacitor. In terms of the MnO_2_–MWCNT//Zn ZIHSC hybrid device, the electrochemical measurements and analysis of the cathode active material MnO_2_–MWCNT for galvanostatic charge–discharge (GCD) demonstrate a maximum specific capacity of 339.98 mAh/g and MnO_2_ delivers a specific capacity of 203.52 mAh/g at 0.2 mA/g. Additionally, electrochemical impedance spectroscopy (EIS) analysis shows the enhanced conductivity and lower charge transfer resistance of the MnO_2_–MWCNT electrode, indicating efficient ion and electron transport.

A key contributing factor is the use of a semi-solid gel electrolyte, which facilitates improved ion transport compared to conventional aqueous systems and enhances cycling stability. As a result, the device exhibits a high coulombic efficiency of 99.5% and a capacity retention of 94.15%, along with excellent cycling performance. Overall, the Zn–Mn-based hybrid device demonstrates superior energy storage capability, where the MnO_2_–MWCNT device realizes a good specific energy storage system. In conclusion, the integration of battery-type MnO_2_ nanorod cathodes with a conductive and hydrophilic functionalized MWCNT network provides a promising pathway for the progress of advanced aqueous rechargeable energy storage devices for future applications.

## Figures and Tables

**Table 1:** Binding energy (BE) positions, fitted Mn 2p chemical states, and corresponding atomic percentages from the XPS deconvolution analysis.

BE Position	‘Mn 2p’ Fitted Composition State	Atomic Percentage
641.19 eV	Mn2p^3/2^ (Mn^3+^)	32.01%
642.87 eV	Mn2p^3/2^ (Mn^4+^)	23.03%
646.33 eV	Sat. peak	21.76%
653.57 eV	Mn2p^1/2^ (Mn^3+^)	17.25%
654.49 eV	Mn2p^1/2^ (Mn^4+^)	5.96%

## Data Availability

The original contributions presented in this study are included in the article/Supplementary Material. Further inquiries can be directed to the corresponding author.

## Supplementary Materials

The following supporting information can be downloaded at: https://www.mdpi.com/article/10.3390/polym18182184/s1, Figure S1: FE-SEM image showing the surface morphology of MnO_2_ nanostructures; Figure S2: (a,b) FE-SEM images of as-synthesized functionalized MWCNT material; Figure S3: Raman spectra of (a) MnO_2_, (b) f-MWCNT, and (c) MnO_2_-MWCNT nanocomposite; Figure S4: Comparative plot of specific capacity versus a function of different current densities; Table S1: EIS-fitted Randles equivalent-circuit parameters of the fresh MnO_2_//Zn ZISHC device electrode; Table S2: EIS-fitted Randles equivalent-circuit parameters of the MnO_2_//Zn ZISHC device electrode after cycling; Table S3: EIS Z-fitting parameters of the fresh MnO_2_–MWCNT//Zn ZISHC device electrode; Table S4: EIS Z-fitting parameters of the MnO_2_–MWCNT//Zn ZISHC device electrode after cycling; Table S5: Electrochemical performance evaluation comparison with recently reported state-of-the-art Zn-ion hybrid capacitor devices [60,61,62,63,64,65].
