# Supplementary material for: Developing Polymer Semi-Solid-State Gel Electrolyte with High-Performance Aqueous Zn-Mn Battery-Type Hybrid Capacitor Device for MnO2–MWCNT Cathode
Source: Polymers (Basel). 2026 Sep 8;18(18):2184. doi: 10.3390/polym18182184 (PMC13610513; doi:10.3390/polym18182184)
Supplement: Supplementary file 1 [file polymers-18-02184-s001.zip › polymers-4432744-supplementary.pdf]

Developing polymer semi-solid-state gel electrolyte with High-Performance Aqueous Zn-Mn Battery type Hybrid Capacitor device for MnO_2@f-MWCNTs Cathode.

Vediyappan Thirumal[1], Perumal Rajivgandhi[2], Jinho Kim*[1]

[1]School of Mechanical Engineering, Yeungnam University, Gyeongsan, 38541, South Korea

[2]Department of Chemistry, Nehru Memorial College (Affiliated to Bharathidasan University), Puthanampatti, Trichy 621 007, Tamil Nadu, India.

*Correspondence: jinho@ynu.ac.kr (JHK)

Supporting information

Specific capacity and Energy-power density calculation:

The charge-discharge profile data from these tests were analyzed, and specific capacitance was observed from GCD profile using the following formula (Eq. (S1)):

$$Qs = \frac{I \times \Delta t}{3.6 \times m} \text{ mAh/g} \quad - \quad \text{Eq. - (S1)}$$

Where Eq. (1) Qs (mAh/g) is the specific capacitance, I (mA) is the charge-discharge current, Δt (s)-discharging time, (m-mg) is the mass of specific active material.

The power and energy density calculation used for the MnO_2-MWCNT//Zn hybrid battery device was calculated via the formula Eq-S2 and Eq-S3,

$$ED = \frac{Qs \times \Delta V^2}{7.2} \text{ Wh/kg} \ - \ \text{Eq. - (S2)}$$

$$PD = \frac{3600 \times ED}{\Delta t} \text{ W/kg} \ - \ \text{Eq. - (S3),}$$

ED = specific energy density (Wh/kg), PD = specific power density (W/kg), discharge time (Δt(s)), total potential window ΔV (V) and specific capacitance Qs (mAh/g).

FE-SEM surface morphology images

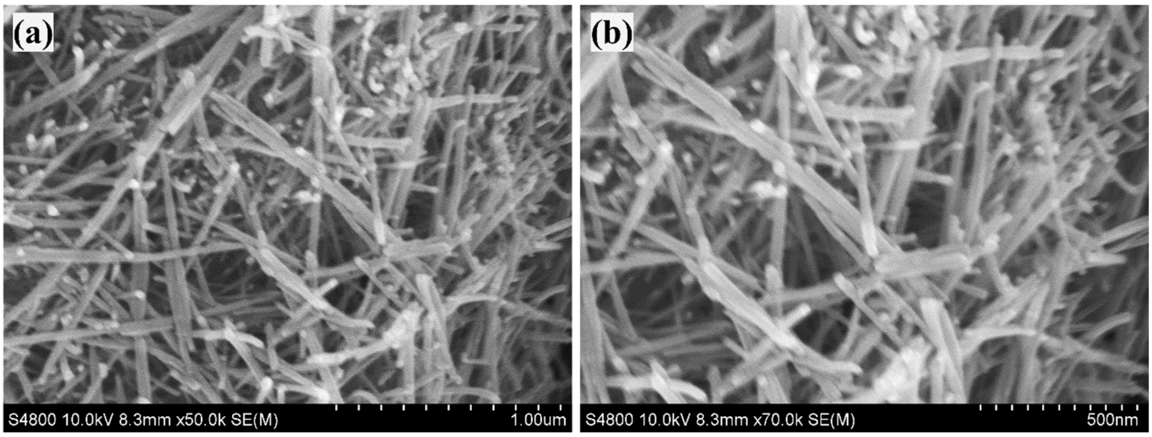

Figure S1 (a, b) FE-SEM image showing the surface morphology of MnO_2 nanostructures

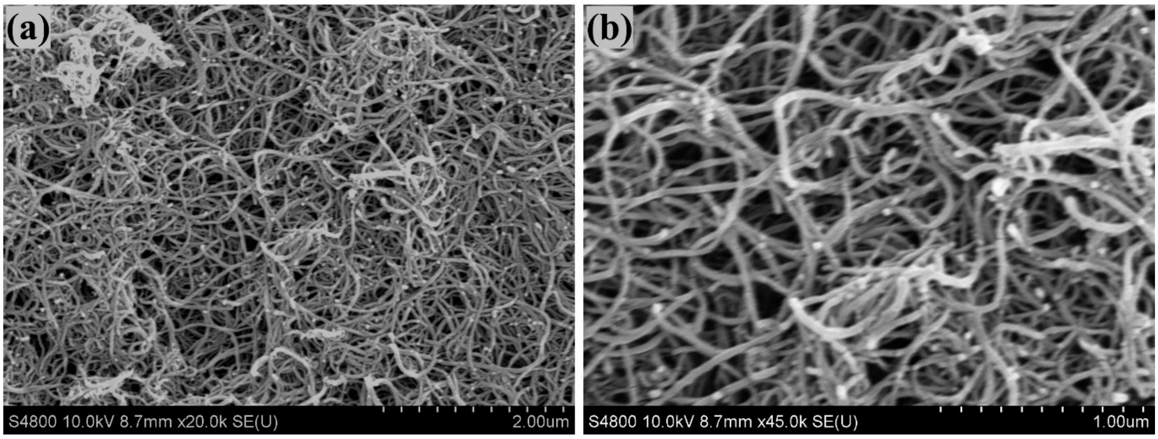

Figure S2 (a, b) FE-SEM images of as synthesized functionalized MWCNTs material

3.4 RAMAN Studies

Raman spectroscopy was employed to analyze the bonding nature and structural features of the as-prepared MnO_2 nanorods, functionalized MWCNTs, and MnO_2–MWCNTs nanocomposites are presents in Fig. S3. The pure MnO_2 sample exhibits characteristic Raman peaks at 107.38, 145.57, 256.72, and 609.55 cm-1, which are attributed to lattice vibrational modes of Mn–O bonds, confirming the formation of well-crystallized manganese oxide nanorods [60]. These low-

frequency modes are associated with Mn–O bending and stretching vibrations within the MnO_6 octahedra. The functionalized MWCNTs show two prominent peaks at 1343.13 cm^{-1} (D-band) and 1579.11 cm^{-1} (G-band), corresponding to disordered carbon defects and in-plane vibration of sp^2-bonded carbon, respectively.

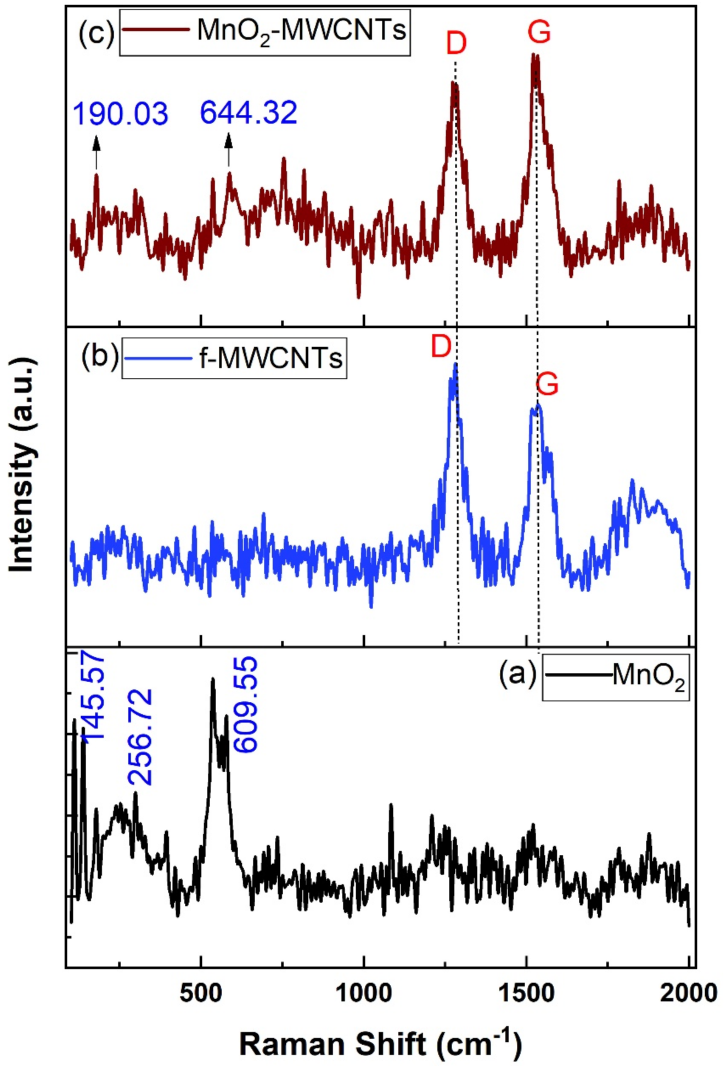

Figure S3. Raman spectra (a) MnO2, (b) f-MWCNTs and (c) MnO_2-MWCNTs nanocomposite, (Inset: Raman bands denotes D & G; D band-disorder band; G band-graphitic band).

The calculated intensity ratio (I_D/I_G = 0.86) indicates the presence of structural defects introduced by surface functionalization, mainly due to carboxyl and hydroxyl groups on CNT walls. For the

MnO_2–MWCNTs nanocomposite materials, for manganese dioxide (MnO_2) distinct Raman peaks appear at 190.03 and 644.32 cm^{-1}, matching to Mn–O vibrational modes and confirming the retention of MnO_2 structure in the composite [61]. Additionally, the CNT-related D- and G-bands are observed at 1346.55 cm^{-1} and 1581.39 cm^{-1} with good intensity. The slightly increased disorder ratio (I_D/I_G = 0.93) suggests enhanced interfacial interaction between MnO_2 nanorods and CNTs after hydrothermal anchoring. Overall, the Raman results confirm the successful integration of MnO_2 nanorods with functionalized CNTs, forming an electrochemically active composite suitable for energy storage applications.

Electrochemical Capacity Rate Capability Behavior vs. Current Density:

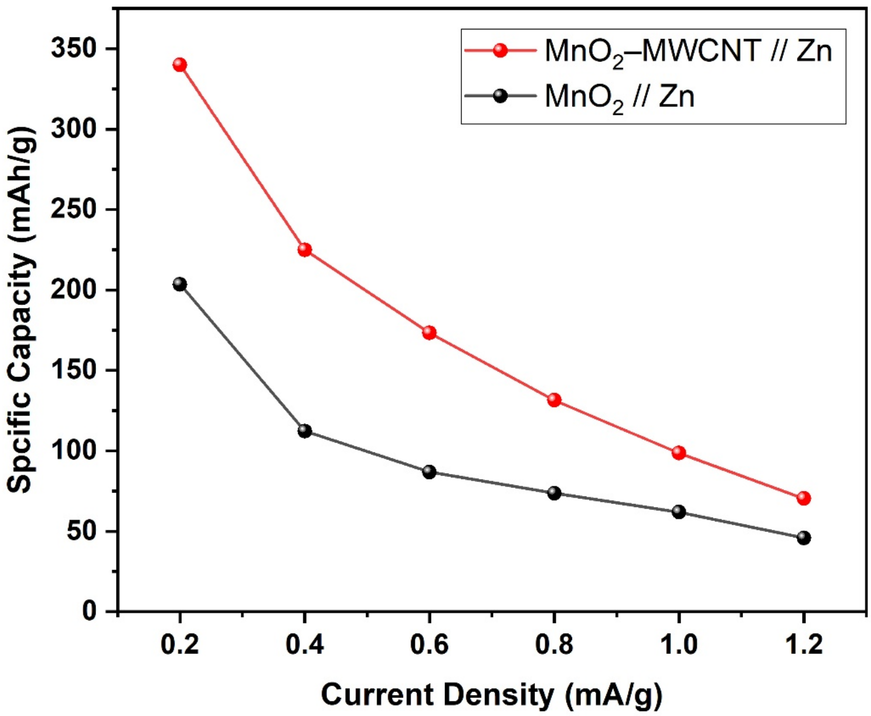

Figure S4. Comparative plot of specific capacity versus a function of different current densities.

Table S1; EIS-fitted Randles equivalent-circuit parameters of the fresh MnO_2//Zn ZIHC device electrode.

**Randles circuit element**	**Fitted circuit values**
R1	6.67 Ohm
R2	8.09 Ohm
CPE (Q1)	7.652582203 e^{-6} F.$s^{(a-1)}$
W1	17.30632699 Ohm.$s^{-1/2}$

Table S2; EIS-fitted Randles equivalent-circuit parameters of the MnO_2//Zn ZIHC device electrode after cycling.

**Randles circuit element**	**Fitted circuit values**
R1	R1 = 4.55 Ohm
R2	12.178 Ohm
CPE (Q1)	3.444832991e^{-6} F.$s^{(a-1)}$
W1	9.686047476 Ohm.$s^{-1/2}$

Table S3; EIS Z-fitting parameters of the fresh MnO_2–MWCNT//Zn ZIHC device electrode.

**Randles circuit element**	**Fitted circuit values**
R1	5.42 Ohm
R2	6.09 Ohm

CPE (Q1)	Q2 = 92.950002e^{-6} F.s$^{(a-1)}$
W1	3.748 Ohm.s$^{-1/2}$

Table S4; EIS Z-fitting parameters of the MnO_2–MWCNT//Zn ZIHC device electrode after cycling.

**Randles circuit element**	**Fitted circuit values**
R1	5.23 Ohm
R2	7.6 Ohm
CPE (Q1)	51.49253731e^{-6} F.s$^{(a-1)}$
W1	5.8999878 Ohm.s$^{-1/2}$

Table S5; Electrochemical performance evaluation comparison with recently reported state-of-the-art Zn-ion hybrid capacitors devices.

**Electrode / Device Configuration**	**Specific Capacity (mAh/g**	**Energy Density (Wh/kg)**	**Capacity Retention**	**Cycling Stability**	**Year**	**Ref.**
MnO_2–CNTs // $Ti_3C_2T_x$ MXene ZIHC	~115.1 [*]	98.6	83.6%	15,000 cycles	2019	[62]
CNT@MnO_2 // Zn	105.6	-	85.72%	1000 cycles	2021	[63]
$Ti_3C_2T_x$ aerogel // Zn	233.5	129.83	98.3%	5000 cycles	2023	[64]
CNTs@MnO_2 // Nb_2CT_x MXene ZIHC	-	96.7	88.7%	10,000 cycles	2024	[65]

RGO-V_2O_5 // RGO-$Ti_3C_2T_x$ ZIHC	175	107.2	81%	10,000 cycles	2026	[25]
**MnO2-f-MWCNTs // Zn**	**339.98**	**157.71 1.0 mA/g**	**94.15%**	**15,000 cycles**	**2026**	**This work**

*Values reported as specific capacitance (F/g)

References:

[60] T. Gao, H. Fjellvag, P. Norby, A comparison study on Raman scattering properties of α- and β-MnO2, Analytica Chimica Acta, 648 (2009) 235-239, https://doi.org/10.1016/j.aca.2009.06.059

[61] J. E. Post, D. A. McKeown, P. J. Heaney, Raman spectroscopy study of manganese oxides: Layer structures, American Mineralogist (2021) 106 (3): 351–366, https://doi.org/10.2138/am-2021-7666

[62] S.Wang, Q. Wang, W. Zeng, M. Wang, L. Ruan, Y. Ma., A New Free-Standing Aqueous Zinc-Ion Capacitor Based on MnO2–CNTs Cathode and MXene Anode. Nano-Micro Lett. 11, 70 (2019). https://doi.org/10.1007/s40820-019-0301-1

[63] Zuo, L.; Sun, H.; Yuan, X.; Wen, J.; Chen, X.; Zhou, S.; Wu, Y.; van Ree, T. Agar Acts as Cathode Microskin to Extend the Cycling Life of Zn//α-MnO2 Batteries. Materials 2021, 14, 4895. https://doi.org/10.3390/ma14174895

[64] A. Mateen, M. Z. Ansari, Ti2CTx–MXene aerogel based ultra–stable Zn–ion supercapacitor, Composites Communications, 38, 2023, 101493., https://doi.org/10.1016/j.coco.2023.101493

[65] Z. Zou, H. Chen, Q. Li, Y. Wei, X. Zhang, X. Zou, X. Mei., Excellent Zinc-Ion Hybrid Capacitor Based on the Nb2CTx Anode and CNTs@MnO2 Nanocomposite Cathode, ACS Appl. Nano Mater. 2024, 7, 17, 20645–20652, https://doi.org/10.1021/acsanm.4c03621

[25] S. De, B. P. Bastakoti, Bimetallic (Co/Ni, Ce) MOF decorated V2CTx MXene/CNT for high energy flexible zinc-ion capacitor, J. Mater. Chem. A, 2026,14, 7407-7420, https://doi.org/10.1039/D5TA08281A
